# Structural and functional characterization of USP47 reveals a hot spot for inhibitor design

**DOI:** 10.1038/s42003-023-05345-5

**Published:** 2023-09-22

**Authors:** Sang Chul Shin, Jinyoung Park, Kyung Hee Kim, Jung Min Yoon, Jinhong Cho, Byung Hak Ha, Yeonji Oh, Hyunah Choo, Eun Joo Song, Eunice EunKyeong Kim

**Affiliations:** 1 Biomedical Research Institute, Korea Institute of Science and Technology, Seoul, 02792 Republic of Korea; 2 Division of Bio‑Medical Science and Technology, KIST‑School, University of Science and Technology (UST), Seoul, 02792 Korea; 3 Graduate School of Pharmaceutical Sciences, College of Pharmacy, Ewha Womans University, Seoul, 03760 Republic of Korea; 4 Brain Science Institute, Korea Institute of Science and Technology, Seoul, 02792 Republic of Korea; 5 Present Address: Research Resources Division, Korea Institute of Science and Technology, Seoul, 02792 Republic of Korea; 6 Present Address: Dana-Farber Cancer Institute, Boston, MA 02215 USA

**Keywords:** X-ray crystallography, Enzyme mechanisms

## Abstract

USP47 is widely involved in tumor development, metastasis, and other processes while performing a more regulatory role in inflammatory responses, myocardial infarction, and neuronal development. In this study, we investigate the functional and biochemical properties of USP47, whereby depleting USP47 inhibited cancer cell growth in a p53-dependent manner—a phenomenon that enhances during the simultaneous knockdown of USP7. Full-length USP47 shows higher deubiquitinase activity than the catalytic domain. The crystal structures of the catalytic domain, in its free and ubiquitin-bound states, reveal that the misaligned catalytic triads, ultimately, become aligned upon ubiquitin-binding, similar to USP7, thereby becoming ready for catalysis. Yet, the composition and lengths of BL1, BL2, and BL3 of USP47 differ from those for USP7, and they contribute to the observed selectivity. Our study provides molecular details of USP47 regulation, substrate recognition, and the hotspots for drug discovery by targeting USP47.

## Abstract (web summary)

Crystal structures of USP47’s catalytic domain in its free and ubiquitin-bound states and functional assays reveal molecular details of USP47 regulation, substrate recognition, and hotspots for drug discovery.

## Introduction

Ubiquitin-specific protease 47 (USP47), a member of the USP family of deubiquitinating enzymes (DUBs), is involved in diverse cellular processes. Following the identification of USP47 as an interacting protein of β-TrCP^1^, various functions such as neuronal development, viral entry, inflammation, and more have been reported. USP47 regulates katanin p60-mediated axonal growth with the C-terminus Hsp70-interacting protein (CHIP)^2^. Furthermore, USP47 knockdown prevents the proper entry of the influenza virus into the host cell^3^. USP47 also has a role in inflammation by regulating inflammasome activation and the release of proinflammatory cytokines, such as IL-1β and IL-18^4^. Moreover, many studies have shown that USP47 exhibits critical functions in cell growth and genome integrity that are closely related to cancer. Bufalieri *et al*. identified endoplasmic reticulum aminopeptidase 1 (ERAP1) as a new player in the Hedgehog pathway, and ERAP1 binds to USP47, displacing β-TrCP from SCF^β-TrCP^, a ubiquitin ligase, therefore promoting β-TrCP degradation^5^. Also, Yu et al. reported a reversible regulation of SATB2 ubiquitination by USP47 and SMURF2, mediating colon cancer cell proliferation and tumor progression^6^. USP47 is a direct target of miR-188-50, and the overexpression of USP47 attenuated LINC00669 knockdown-induced tumor-suppressive effects in colorectal cancer cells^7^. Furthermore, we found that USP47 can function as a regulator of the MDM2-p53 axis by deubiquitinating ribosomal protein S2 (RPS2), e.g., ribosomal stress decreased the interaction between RPb2 and USP47, causing RPS2 to disassociate and inhibit MDM2, which subsequently lead to p53 stabilization. We further confirmed that USP47 could deubiquitinate RPL11, another ribosomal protein, controlling the activity of p53 and apoptosis in cancer cells by regulating the localization of RPL11 and its interaction with MDM2^8^. The depletion of USP47 using siRNA or miRNA inhibited cancer cell growth and colony formation in a p53-dependent manner^8,9^. In addition, inhibition of USP47 has been suggested as a novel targeted therapy to overcome resistance to tyrosine kinase inhibitors in chronic myelogenous leukemia (CML)^10^ and hematologic malignancies expressing mutant EZH2^11^. USP47 knockdown significantly inhibited both BCR-ABL-induced CML in mice and BCR-ABL^T315I^-induced CML, which are known to confer Imatinib and second-generation TKI resistance^10^. USP47 inhibition blocked the function of mutated EZH2 through ubiquitination and EZH2 degradation, increasing the sensitivity of diffuse large B-cell lymphoma (DLBCL) and acute myeloid leukemia (AML) cells to EZH2 inhibitors, such as the recently FDA-approved EPZ-6438, in vitro and in vivo^11^.

These studies all demonstrate the significance of USP47 as a therapeutic target and the importance of developing USP47 inhibitors. However, no specific USP47 inhibitors have currently been developed, although first-generation USP7 inhibitors, such as P5091 (1-[5-(2,3-dichloro phenylsulfanyl)−4-nitro-2thien l]ethanone) have been shown to inhibit both USP7 and USP47 with an IC_50_ of 4.2 µM and 4.3 µM, respectively, and antitumor efficacy in both in vitro and in vivo multiple myeloma xenograft models^12,13^. Parthenolide, a natural sesquiterpene lactone, can also inhibit both DUBs^14,15^. USP7 is a promising target for cancer therapy as its inhibition is expected to decrease the function of oncogenes, tumor suppressor function, and enhance immune function. Subsequently, extensive drug discovery has been ongoing^16–21^. The catalytic domain in USPs usually comprises about 350 amino acids and adopts a conserved right-handed architecture with “Fingers, Palm, and Thumb” subdomains, where an antiparallel β-sheet structure provides a deep cleft for ubiquitin-binding, with the catalytically active Cys box, and a His box located at the poising sides of the “Palm and Thumb” regions. For USP7, during a catalytic reaction, the incompetent catalytic domain gets realigned, whereby the active site undergoes dramatic conformational changes upon the binding of Ub^22,23^, unlike other DUBs whose structures are reported.

Some biological roles performed by USP47 are similar to those by USP7, although the modulators that control the mechanistic signaling by the two DUBs may differ. Furthermore, both DUBs are closely related phylogenetically. All these findings prompted us to investigate the biochemical and structural properties of USP47, which will provide the ground to assist in developing specific inhibitors. Firstly, we examined the functionality of USP47 in cancer cell proliferation and characterized its biochemical properties. Next, we determined the crystal structures of the USP47 catalytic domain in isolation and the Ub-bound state and carried out structure-based mutational studies to understand the enzymatic mechanism.

## Results

### Cancer cell growth is inhibited more effectively by co-depletion of USP47 and USP7 than by depletion of either one alone

**Fig. 1:**
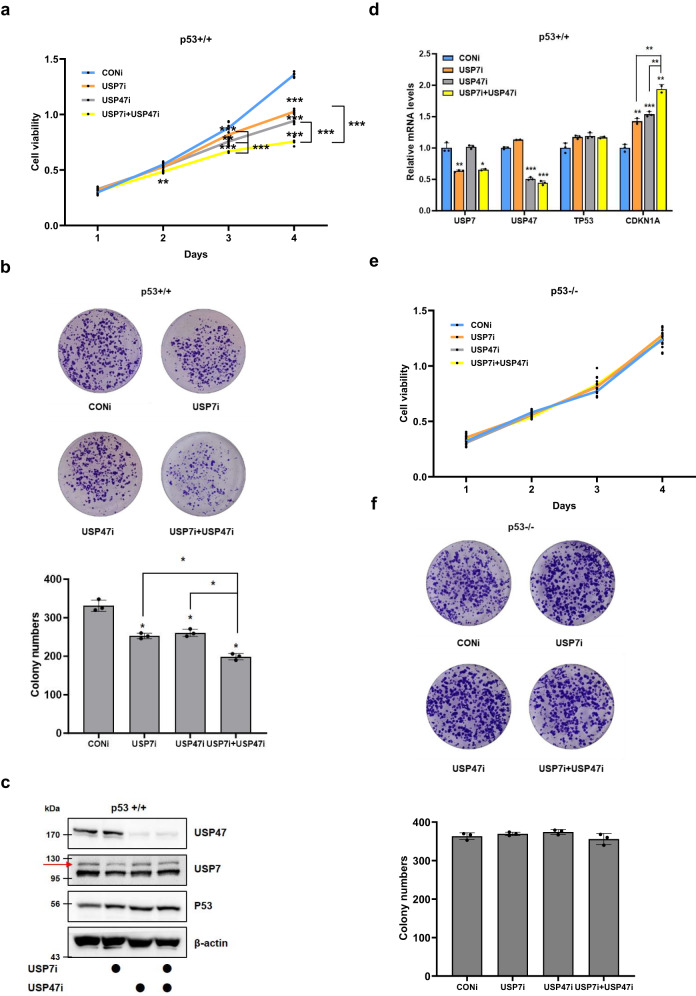
Functional properties of USP47. **a**, **b** HCT116 (p53+/+) cells were transfected with siRNA targeting USP7 or USP47, either alone or together. **a** Cell viability was measured by WST-1 assay at the times indicated. **b** For the colony formation assay, cells were cultured for 10 days and stained with Crystal violet. Stained colonies were counted in three independent experiments. **c**, **d** HCT116 (p53+/+) cells were transfected with siRNA targeting USP7 or USP47, either alone or together. **c** Western blot analysis was performed to detect indicated protein levels. **d** Q-PCR analysis was conducted to determine the relative expression levels of indicated genes. **e**, **f** HCT116 (p53−/−) cells were transfected with siRNA targeting USP7 or USP47, either alone or together. **e** Cell viability was measured by WST-1 assay at the times indicated. **f** For the colony formation assay, cells were cultured for 10 days and stained with Crystal violet. Stained colonies were counted in three independent experiments. The data represent the mean ± SD (*: *p* < 0.05, **: *p* < 0.005, and ***: *p* < 0.0005).

Previous studies have reported that depletion of USP7 or USP47 inhibits cancer cell growth^9,24^. While some substrates are shared by USP47 and USP7, not all are affected by both enzymes. Therefore, we conducted a comparative analysis to assess the impact of co-depletion of USP7 and USP47 versus individual depletion on cancer cell growth. To investigate this, we transfected p53-positive (p53+/+) and p53-deficient (p53−/−) HCT116 cells with siRNAs targeting USP7 (USP7i), USP47 (USP47i), or both (Supplementary Figure 1a–d). Initially, we evaluated cell viability using WST-1 assays (Fig. 1a). Four days post-transfection, we observed a significant reduction in cell viability upon co-transfection of USP7i or USP47i in comparison to transfection with each siRNA alone, specifically in p53-positive HCT116 cells (Fig. 1a). Subsequently, we examined the effects of co-depleting USP7 and USP47 on colony formation. The number of colonies decreased by approximately 30% in USP7 or USP47-reduced cells and by about 50% in cells where both USP7 and USP47 were reduced, relative to control cells (Fig. 1b). Given the association of USP7 and USP47 with the MDM2-p53 pathway^9,25^, we investigated whether the impact of USP7 and USP47 on cancer cell growth is dependent on p53. Knockdown of USP7 or USP47 increased the protein levels of p53 and the mRNA levels of *CDKN1A*, a p53 target gene encoding p21 protein, without affecting p53 gene expression (Figs. 1c, d). In p53-deficient HCT116 cells, transfection of USP7i or USP47i alone did not yield any discernible differences in cell viability or colony formation compared to control cells. Additionally, co-transfection of USP7i and USP47i into p53-deficient HCT116 cells did not alter cancer cell growth (Fig. 1e, f). Therefore, these effects of USP7 and USP47 occur in a p53-dependent manner. Collectively, these results illustrate the non-redundancy of USP47 compared to USP7 by showing that co-depletion of USP7 and USP47 using siRNA co-transfection results in a more significant reduction of cancer cell growth compared to depleting each independently.

### USP47 UBL domains enhance the DUB activity of the USP47 catalytic domain

**Fig. 2:**
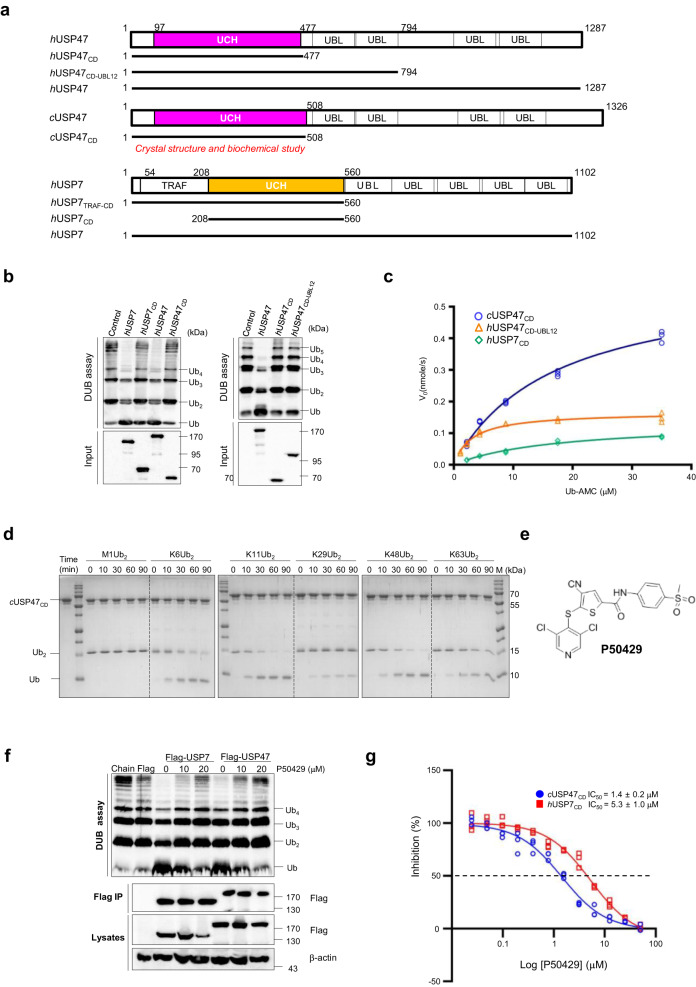
Biochemical properties of USP47. **a** Domain structure of USP47 and the constructs used in this study. **b** HEK293T cells were transfected with USP Flag-tagged mutants. After the immunoprecipitation with anti-Flag affinity gel, K48-linked ubiquitin chains were incubated with immunoprecipitated Flag-USP7 or Flag-USP47 at 30 °C for 90 min. Each cleaved ubiquitin chain was detected by immunoblotting with an anti-ubiquitin antibody. **c** Michaelis–Menten plot for *h*USP47_CD_-_UBL12_ (in 100 nM), *c*USP47_CD_ (in 50 nM), and *h*USP7_CD_ (in 100 nM). **d** Gel-based cleavage assay of the *c*USP47_CD_ using M1-, K6-, K11-, K29-, K48-, and K63-diUb substrate. **e** Chemical structure of dual inhibitor P50429. **f** HEK293T cells were overexpressed with Flag-USP7 or Flag-USP47, and then, treated with DMSO or the indicated concentration of P50429 for 2 h. After the immunoprecipitation with anti-Flag affinity gel, K48-linked ubiquitin chains were incubated with immunoprecipitated Flag-USP7 or Flag-USP47 at 30 °C for 90 min. The reactions were stopped, and the cleaved ubiquitin chains were detected by immunoblotting with an anti-ubiquitin antibody. **g** Inhibition kinetics in *c*USP47_CD_ and *h*USP7_CD_. The IC_50_ values were determined in triplicate and fitted to a four-parameter logistic dose-response curve. Data are expressed as means ± SD.

As shown in Fig. 1, USP47 and USP7 inhibit cancer cell growth in a p53-dependent manner. Next, we tested whether the activity of USP47 is also similarly regulated since the catalytic activity of USP7 is tightly regulated by the UBL domains and the C-terminal residues^18,26,27^. In particular, the last two UBL domains in USP7 promote ubiquitin binding and increase USP7 DUB activity by 100-fold. Thus, to identify whether the USP47 UBL domains can also affect the DUB activity, we designed several constructs, shown in Fig. 2a.

Most of the constructs for human USP47 (*h*USP47) were practically insoluble despite all our efforts, similar to other USPs^28^, except for the USP47 catalytic domain from *C. elegans* (*c*USP47_CD_, residues 1–508) and *h*USP47_CD-UBL12_ (1-794) (Supplementary Figure 2). However, using cell lysates, we could confirm that both the full-length (*h*USP47, 1–1287) and the USP47 catalytic domain (*h*USP47_CD_, 1–477) cleaved the K48-linked Ub chain to mono-Ub, while the full-length enzyme was more active than in the catalytic domain alone, which was similar to the data for USP7 (Fig. 2b, left). In addition, the catalytic activity of *h*USP47_CD-UBL12_ was slightly higher than for the catalytic domain alone (Fig. 2b, right). These results suggest that the C-terminal UBL domains of *h*USP47_CD-UBL12_ are similar to USP7 in that they support the DUB activity of the USP47 catalytic domain. Using the obtained *h*USP47_CD-UBL12_ and *c*USP47_CD_ recombinant proteins, we measured their catalytic activity using Ub-AMC. For comparison, *h*USP7_CD_ was used as a reference, although it possesses low DUB activity compared to the full-length enzyme.

The Michaelis–Menten analysis provided a *K*_M_ = 3.0 ± 0.6 μM and *k*_cat_ = 0.03 ± 0.002 s^−1^ for *h*USP47_CD-UBL12_ and values of 16.8 ± 3.42 μM and 0.2 ± 0.02 s^−1^ for *c*USP47_CD_ (Fig. 2c and Supplementary Fig. 2). The resulting catalytic efficiencies (*k*_cat_/*K*_M_) of 12 × 10^3^ M^−1^ s^−1^ and 10 × 10^3^ M^−1^ s^−1^, respectively, are higher than for *h*USP7_CD_ (3 × 10^3^ M^−1^ s^−1^). The reported value for *h*USP7_CD_ was 4 × 10^3^ M^−1^ s^−1^^26^. Then, we tested whether USP47 demonstrated any chain specificity using various di-ubiquitin chains. As seen in Fig. 2d, *c*USP47_CD_ showed slightly higher DUB activity for K11- or K48-linked di-Ub chains than for the K6-, 29-, and 63-linked di-Ub chains. Next, we tested the effect of P50429 (Fig. 2e), which is a P5091 derivative^29^. Expectedly, P50429 inhibited the DUB activity of both *h*USP7_CD_ and *c*USP47_CD_ (Fig. 2f). To elucidate the effect of P50429 in vitro, we measured the IC_50_ of *c*USP47_CD_ and *h*USP7_CD_ (Fig. 2g). The IC_50_ values were 1.4 ± 0.2 and 5.3 ± 1.0 μM, respectively, indicating that P50429 can inhibit the catalytic domain of both *c*USP47_CD_ and *h*USP7_CD_ in vitro.

### Ub-binding prompts the activation of misaligned catalytic triads for catalysis

**Fig. 3:**
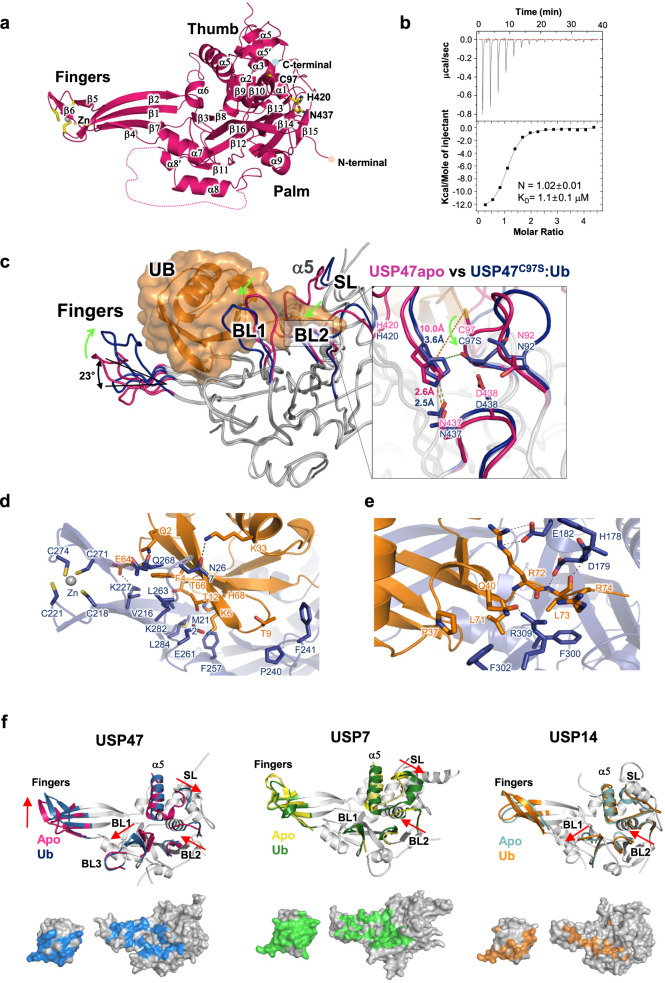
Crystal structures of the USP47 catalytic domain with and without bound Ub. **a** Ribbon representation of USP47 catalytic domain from *C. elegans*. The Fingers, Palm, and Thumb subdomains are indicated with the Zn ion shown as a yellow sphere. **b** ITC binding curve for Ub to *c*USP47_CD_^C97S^ catalytic domain. The lower panel shows the integrated heat data zagainst the molar ratio of Ub to *c*USP47_CD_^C97S^. The closed squares were fitted to a one-site model, the solid lines represent the best-fit results, and the error in the *K*_D_ values corresponds to the standard error of fitting a singlicate dataset. **c** The structure of USP47^C97S^:Ub. USP47^C97S^ and Ub are shown in Cα drawing (gray) and surface presentation (orange), respectively, with the Fingers, BL1, BL2, and SL regions highlighted in blue. The structure of USP47 alone is superimposed on the USP47^C97S^:Ub, and the highlighted regions are shown in maroon. Close-up view at the interface between USP47^C97S^ (blue) and Ub (orange) in the USP47^C97S^:Ub complex structure. **d** at the Fingers region, and **e** at the active site. **f** Comparison of the Ub-bound and apo structures of USP47, USP7, and USP14. Superimposition of the SL region of both USP47 (magenta) and USP47^C97S^ (blue):Ub (light yellow) (left), both USP7 (yellow) and USP7 (green):Ub (light yellow) and both USP14 (cyan) and USP14 (orange):Ub (light orange). PDB entries used are 1NBF and 1NB8 for USP7 and 2AYN and 2AYO for USP14. Below are the interaction surfaces of Ub and USPs (USP7, USP14, and USP47) in the complexes. Residues within 5 Å of each other are colored.

We obtained crystals for *c*USP47_CD_ and determined the crystal structure at 2.6 Å resolution (Table 1 and Supplementary Figure 3). The three molecules in an asymmetric unit have pairwise RMSD ≤ 0.65 Å for 316 Cα atoms. It revealed a canonical USP fold with three distinct subdomains: Thumb, Fingers, and Palm. The Thumb consisted of six α-helices (Fig. 3a). The Fingers comprised four β strands in the center (β1, β2, β4, and β7), an α−helix (α7), and an additional two β strands (β5 and β6) at the tip that harbors four cysteine residues, which coordinates the structural Zn. The Palm connecting the Fingers and the Thumb forms an antiparallel β-sheet (β3, β8, and β16) with four α-helices (α8−α11). While the sequence identity between USP47 orthologues in different species is high (~95% amongst vertebrates), the sequence identity between humans and *C. elegans* is only 44% with four insertions and a deletion (Supplementary Figure 3), which are located mainly in the disordered region away from the catalytic site (>25 Å from the catalytic Cys). Therefore, the overall structure will be the same. Furthermore, the catalytic site, the catalytic loops, and the Ub-binding region are highly conserved, suggesting that they can serve as a basis for understanding the biochemical and structural approach to chemical studies.

**Table 1:** Statistics on the data collection and refinement.

	*c*USP47_SeSAD	*c*USP47_CD_	*c*USP47_CD_^C97S^:Ub
*Data collection*			
Beam Line		PLS BL-5C	
Wavelength, Å	0.97945 (Se-Peak)	1.00000	1.00000
Space group	*C*222_1_	*C*2	*P*2_1_2_1_2_1_
Unit cell parameters			
a, b, c (Å)	91.50; 181.58; 237.42	94.07; 58.90; 251.93	46.95; 93.40; 217.76
α, β, γ (°)	90.0; 90.0; 90.0	90.0; 96.69; 90.0	90.0; 90.0; 90.0
^a^Resolution, Å	50-3.20 (3.31-3.20)	50-2.60 (2.69-2.60)	50-3.00 (3.11-3.00)
No. of total reflections	1,785,413	1,145,218	637,329
No. of unique reflections	33,298	43,015	19,938
No. in asymmetric unit		3	2
^a^Completeness, %	99.2 (93.7)	96.3 (97.3)	91.5 (84.2)
^a^*I*/σ(*I*)	19.1 (4.2)	15.3 (2.1)	7.4 (2.3)
^a^Redundancy	3.3 (1.9)	2.9 (2.2)	4.2 (2.8)
^b^*R*_merge_, %	4.8 (15.4)	7.9 (32.5)	14.0 (37.4)
^a^CC_1/2_, %	98.3 (85.0)	99.6 (85.7)	95.6 (69.3)
*Refinement*			
Resolution, Å		50-2.60 (2.69-2.60)	50-3.00 (3.11-3.00)
^c^*R*_cryst_/*R*_free_, %		25.8/26.9	24.8/28.4
No. of protein atoms		8257	6773
No. of water molecules		184	0
No. of ligand molecules		2	2
RMSD from ideal geometry:			
Bond lengths, Å		0.002	0.003
Bond angles, °		0.503	0.538
Average *B*-factor, Å^2^		77.6	70.7
Ramachandran analysis			
Favored, %		94.5	95.1
Allowed, %		5.5	4.9
Outliers		0	0
Rotamer outliers, %		1.2	1.5
PDB entry		8ITN	8ITP

^a^Values in parentheses are for the outer most resolution shell.

^b^*R*_merge_ = ∑_h_∑_i_∣*I*(*h*,i) - <*I*(*h*)>∣ / ∑_h_∑_i_ *I*(*h*,i), where *I*(*h*,i) is the intensity of the i^th^ measurement of reflection *h* and <*I*(*h*)> is the mean value of *I*(*h*,i) for all i measurements.

^c^*R*_free_ was calculated from randomly selected 5% set of reflections not included in the calculation of the *R* value.

To obtain the Ub-bound structure without perturbing the active site, we first tested Ub-binding using ITC. The wild type did not show measurable binding (Supplementary Fig. 4). The binding affinity between the wild type USPs and Ub has not been easy, possibly due to the low binding, e.g., 105.7 ± 15.9 μM for USP7^29^, or the intrinsic flexibility in the binding sites. Various mutations in either ubiquitin or USPs have been made to overcome this^30–36^. As such, we mutated the catalytic Cys to Ser and Ala. While the *c*USP47_CD_^C97A^ did not show measurable binding, *c*USP47_CD_^C97S^ showed a 1:1 molar binding with *K*_D_ = 1.1 μM ± 0.1 μM (Fig. 3b and Supplementary Fig. 4), and this mutant led us to a non-covalently Ub-bound structure. For crystallization, we incubated *c*USP47_CD_^C97S^ with Ub before crystallization instead of using Ub-aldehyde or Ub-PA, which results in a covalent adduct to the catalytic Cys. The crystals diffracted to 3.0 Å resolution (Table 1), and the structure was solved using the free *c*USP47_CD_^C97S^ described above. The two *c*USP47_CD_^C97S^:Ub complexes in an asymmetric unit had an RMSD of 0.54 Å for 354 Cα atoms. The resulting map showed bound Ub, although the two Gly residues were disordered at the C-terminus in Ub (Fig. 3c and Supplementary Figure 5). The β-sheet of Ub was packed against the Fingers and Palm regions in *c*USP47_CD_, making extensive interactions (Fig. 3d). On the other hand, the C-terminus of Ub was bound in the narrow cleft between the Palm and the Thumb, leading to the catalytic site (Fig. 3e). Leu71 and Leu73 of Ub make extensive hydrophobic contact with Val180, Phe300, Phe302, and Tyr468 of USP47 and the backbone of Arg72 and Leu73 of Ub forms H-bonds with Asp179 of USP47, in addition to the 2:2 salt bridge made between the guanidinium group of Arg72 of Ub and Glu182 of USP47.

The overall structure of the catalytic domain region of the structural model for human USP47 derived from the alpha fold (AF-Q96K76-F1) was the same as the crystal structures determined here. Interestingly, the model was closer to the crystal structure of the Ub-bound *c*USP47_CD_ than the apo *c*USP47_CD_, although the model did not include Ub (Supplementary Fig. 6). The catalytic residues were in line with the catalysis of the model. However, the loop conformations at the active site differed from those identified in the two crystal structures here. In particular, the BL1 differed significantly, e.g., the Cα–Cα distance at the tip of the BL1 was >10 Å.

Structural changes upon Ub-binding were observed at the catalytic triads, the three loops near the catalytic sites known as the switching loop (SL), blocking loop 1 (BL1), blocking loop 2 (BL2), and the Finger regions (Figs. 3c, f and Supplementary Fig. 7). The RMSD between the apo and Ub-bound structures was 1.2 Å for the 307 Cα atoms. In the apo form, the Sγ of the catalytic Cys97 was 10.0 Å from Nδ1 of His420 (Fig. 3c, insert), although the Nε2 of His420 made a hydrogen bond with Asn437 Oδ1. Therefore, the catalytic triads were incompetent for catalysis. However, in the Ub-bound structure, the N-terminal region of α1 became helical, putting the Oγ of Ser97 (equivalent to Sγ of the catalytic Cys97) 3.6 Å away from His420 Nδ1, thereby all three catalytic residues in their canonical positions. Both BL1 and BL2 were twisted toward Ub in a concerted manner (Fig. 3c, f), with the tip showing significantly larger movements (>8.0 Å). Some of the sidechains shifted to accommodate the Ub, e.g., the sidechains of Phe302 and Met307 in USP47 rotated to create space for the Ub Leu73 sidechain. Furthermore, the Arg309 sidechain, which occupied the entrance to the catalytic channel, moved outward to stabilize the tail of the bound Ub. The SL shifted as much as BL1. Another dramatic change occurs in the Fingers region. The Finger region rolls toward the Palm and Thumb by about 23° from the tip of β4. The α5, α6, and Zn shifted by approximately 4–4.5 Å in the Ub-bound structure. Together, Ub binding appears to induce considerable conformational changes in the SL, BL1, BL2, and Fingers, which positions the Ub and realigns the catalytic residues into place for catalysis.

The structural changes upon Ub-binding to USP47 are similar to those identified for USP7^23,37^ (Fig. 3f and Supplementary Fig. 8), although the changes in the Fingers and BL2 of USP7 are less in magnitude (1.2 and 1.8 Å, respectively). Nevertheless, these changes are significantly different from those observed for USP14^38^, USP28^39,40^, and USP34^41^, the only other USPs for which structures of both Ub-free and Ub-bound (covalently attached to the catalytic Cys) are known. In USP14 and USP28 there is practically no difference in the SL and Fingers, while the tip of the BL1 and BL2 possess slightly larger movements (up to 4.5 Å and 2.3 Å, respectively) upon Ub-binding^38–40^. USP34 shows a similar roll-up movement in the Fingers region toward the catalytic residues by 23°, as in USP47. Also, the catalytic histidine rotated ~180° from the misaligned conformation to the catalytic center in USP34^41^.

The interaction between the USP and Ub can be estimated by the change in the accessible surface areas using the Ub-bound structures. The corresponding value for USP47 is ~3360 Å^2^, similar 3650 Å^2^, 3290 Å^2^, and 3630 Å^2^ of USP7, USP14, and USP34, respectively, but much smaller than 4080 Å^2^ and 4150 Å^2^ seen for USP2^42^ and USP8^43^, respectively. The variation in the surface areas between USPs and Ub may reflect the intrinsic flexibility of USPs in Ub-binding.

### SL rearrangement is necessary to activate catalytic triads

**Fig. 4:**
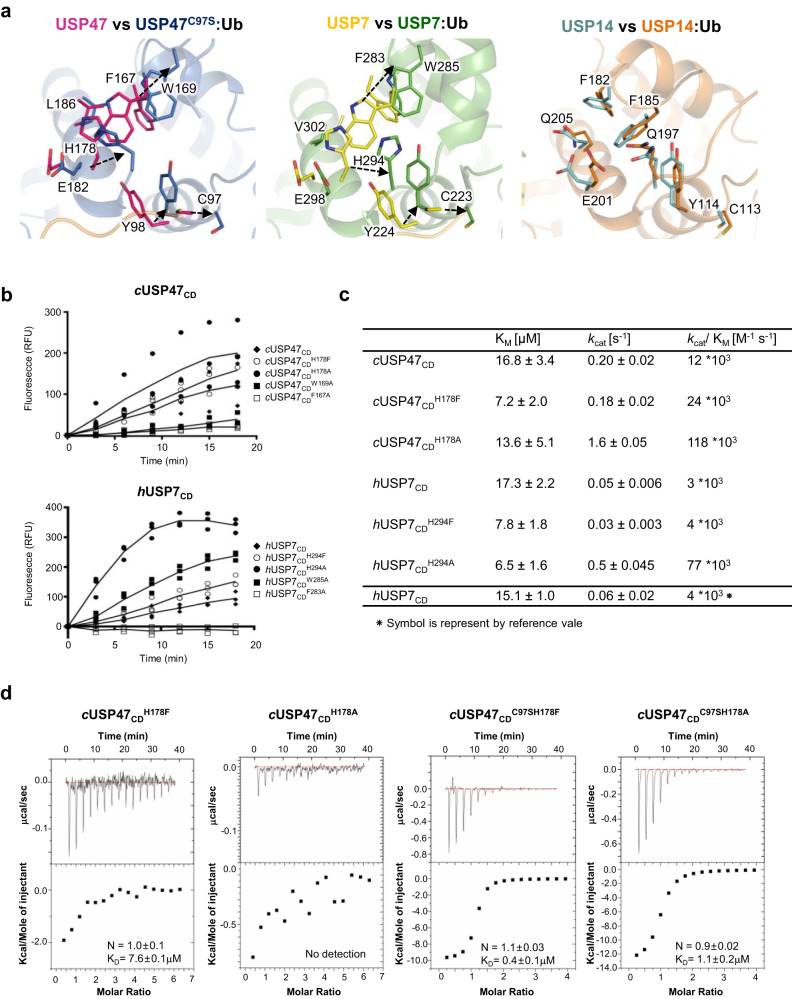
Role of the switching loop (SL) upon Ub-binding. **a** Superimposition of the apo and Ub-bound structures of USP47, USP7, and USP14 at the SL region. Ribbon presentation of USP47 (magenta) and USP7 (yellow; PDB code: 1NB8) and USP14 (cyan; PDB code: 2AYN) alone and in complex with Ub. The Fingers region of USP47 (blue) moves up while the α5 helix of USP7 (green; PBD code: 1NBF) is shifted toward the catalytic triad. There is no significant movement of USP14 (orange; PDB code: 2AY0). **b** (left) Relative activity of the SL mutants of *c*USP47 and *h*USP7. Error-values represent standard deviations for three independent trials. **c** Kinetic analysis of Ub-AMC hydrolysis shows activity for *c*USP47 mutants (H178F and H178A) and *h*USP7 mutants (H294F and H294A). **d** Interactions between USP47 mutants (*c*USP47_CD_^H178F^, *c*USP47_CD_^H178A^, *c*USP47_CD_^C97SH178F^, and *c*USP47_CD_^C97SH178A^, respectively) and ubiquitin were measured using ITC. The error in the *K*_D_ values corresponds to the standard error of fitting a singlicate dataset.

The most dramatic change between the two structures is at the SL in the catalytic site (Figs. 3c and 4a). In the free form, the His178 imidazole ring and the Trp169 indole ring form a π–π interaction, which hovers over a cluster of hydrophobic residues, *e.g*., Tyr98, Leu99, Ile139, Leu163, Phe167, Tyr175, Leu183, Leu186, and Met187 from α1, α5, and SL. The SL takes up a helical segment and puts the Tyr175 sidechain into the hydrophobic cluster. This couples with the rotation of the Tyr98 and the catalytic Cys97 away from the canonical active configuration. In the Ub-bound structure, the catalytic Cys97 and Tyr98 are in active conformations, with the N-terminus of the α1 helix ordered. Alternatively, the SL rearranges, whereby the His178 imidazole ring no longer stacks on the Trp169 indole ring. Another notable change was the Asp179, located on the surface in the free structure. The side chain gets pulled underneath the Ub C-terminal tail to create a hydrogen bond with Tyr421. For USP7, in the absence of ubiquitin, the interactions of the aforementioned hydrophobic cluster are almost identical to those for USP47. The same changes are observed in the Ub-bound USP7 structure, except for in the sidechain of Asp294 (equivalent to Asp179 in USP47), which makes a hydrogen bond with Tyr514 adjacent to Tyr465 (Tyr421 of USP47) (Fig. 4a). However, there was not much change in the position of the catalytic Cys and the SL region in USP14 (Fig. 4a). Ub is covalently attached to the catalytic Cys in USP7^22^ and USP14^38^.

To validate the role of Trp169 and His178 in enzymatic activity, we generated Phe167Ala, Trp169Ala, His178Ala, and His178Phe of *c*USP47_CD_ and tested their enzymatic activity. Furthermore, we constructed the equivalent mutants for *h*USP7_CD_ (Phe283Ala, Trp285Ala, His294Ala, and His294Phe). As expected, these mutants showed a significant increase in the enzymatic activity of both USP47 and USP7, confirming the importance of the hydrophobic cluster in maintaining the orientation of α1, α5, and SL for catalysis (Fig. 4b). In particular, *c*USP47_CD_^F167A^ and *h*USP7_CD_^F283A^ showed practically no activity. We conducted further analysis with the mutations of His178 in *c*USP47_CD_ and His294 in *h*USP7_CD_. Compared to the wild type, both His to Phe mutants showed similar *k*_cat_ values (0.18 ± 0.02 s^−1^ for *c*USP47_CD_ and 0.03 ± 0.003 for *h*USP7_CD_), while the K_*M*_ (7.2 ± 2.0 μM and 7.8 ± 1.8 μM, respectively) showed a significant reduction. Alternatively, in both cases, His to Ala mutation showed a significant increase in *k*_cat_ values (1.6 ± 0.05 s^−1^ for *c*USP47_CD_ and 0.50 ± 0.045 s^−1^ for *h*USP7_CD_), which resulted in higher enzyme efficiency (*k*_cat_/*K*_M_), with approximately 10- and 25-fold increases, respectively (Fig. 4c). Therefore, the π–π interaction between the His178 and Trp169 sidechains in USP47 and His294 and Trp285 in USP7 play a stabilizing role in the inactive conformation found in the two enzymes.

Next, we measured the Ub-binding affinity of these mutants using ITC (Fig. 4d). While *c*USP47_CD_^H178F^ showed a K_*D*_ of 7.6 ± 0.1 μM, His178Ala did not show any significant binding. The double mutants, *c*USP47_CD_^C97SH178F^ and *c*USP47_CD_^C97SH178A^, also demonstrated K_*D*_ values of 0.4 ± 0.1 μM and 1.1 ± 0.2 μM, respectively, which were lower than for *c*USP47_CD_^C97S^ (K_*D*_ = 1.1 ± 0.1 μM). Together, it appears the interaction between Trp169 and His178 (Trp285 and His294 in USP7) in the SL region plays a role in regulating catalytic activity, while Phe167 (Phe283 in USP7) is critical in maintaining the hydrophobic cluster.

### BL1 and BL2 in USP47 differ from those in USP7

**Fig. 5:**
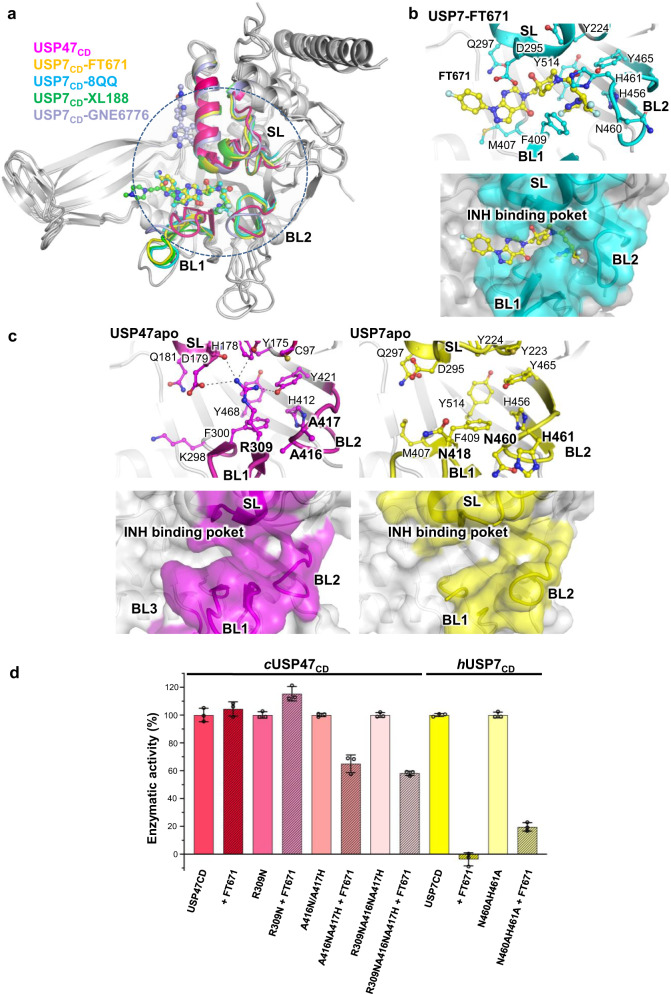
Role of the blocking loops, BL1 and BL2. Comparison of the apo USP47 and inhibitor-bound USP7 structures. **a** Superimposition of *c*USP47_CD_ onto the structures of USP7_CD_ complexed with FT671 (yellow; PBD code: 5NGE), 8QQ (cyan; PDB code: 5N9T), XL188 (green; PBD code: 5VS6), and GNE6776 (gray; PBD code: 5UQX). The SL, BL1, and BL2 are highlighted in the same color as the inhibitors. **b** Close-up of the bound FT671 (yellow) in the USP7–FT671 structure with the molecular surface shown below. FT671 and the key residues in the binding pocket are shown in the stick model. **c** The apo USP47_CD_ and USP7_CD_ (yellow; PDB code: 4M5W) are shown in the stick model (top) and molecular surface presentation (bottom). **d** Relative activity of the BL2 region mutants of *c*USP47_CD_ and *h*USP7_CD_. Kinetic analysis of Ub-AMC hydrolysis shows activity for both *c*USP47_CD_ mutants (^416^Ala–^416^Ala vs. Asn–His) and *h*USP7_CD_ mutants (^460^Asn-^461^His vs. Ala–Ala). Data points are shown as the mean ± SD (*n*  =  3).

In USP7, the residues interacting with P50429, a dual inhibitor, were identified based on the NMR data^29^. Out of the eleven identified USP7 residues, three are different in USP47, i.e., Ala221, Asp289, and Met292 in USP7 are replaced by Met, Glu, and Asp in *c*USP47. In *h*USP47, Asp is Gln, while Met and Glu are conserved, suggesting that the binding surface in USP47 is relatively conserved (Supplementary Figure 3). Since P50429 binds covalently to the catalytic Cys, the residues lining the inhibitor-binding surface are almost the same in the two. Recently reported USP7 inhibitors such as FT671^19^, 8QQ^44^, XL188^17^, and GNE6776^16^ possess high potency and specificity only for USP7 (Fig. 5a). Except GNE6776, most of the USP7 inhibitors target the active site. GNE6776 binds to the Ub-binding surface along the α5 helix of USP7 interacting with Arg301, Asp305, Glu308, Tyr348, Asp349, and His403 of USP7^16^. The charged residues are the same in USP47. However, Tyr348 and His403 are replaced by Leu (Leu232) and Thr (Thr294), respectively (Supplementary Figure 3), therefore suggesting that the reported specificity possibly stems from these differences in the binding surface. The structures of the active site inhibitors complexed to USP7 revealed that the conformation of USP7 in the inhibitor-bound crystal structures is similar to the apo-state. In the case of FT671, the pyrazolo[3,4-*d*]pyrimidin-4-one-piperidine of FT671 interacts with Asp295, Val296, Gln297, Phe409, and Tyr465, while the para-fluorophenyl group extends towards the Finger region (Fig. 5b and Supplementary Fig. 9). The authors reported that the specificity lies in the unique structural features of USP7, which is the rearrangement of the switching loop coupled to the conformational changes of Tyr465 and Tyr514. In USP47, those interacting residues are the same, and the conformational changes in the SL are also the same as those identified in USP7. However, the two Tyr residues in USP47 (Tyr421 and Tyr468) take the same conformation in both the apo- and Ub-bound structures. Further examination of the apo USP47 super positioning onto the FT671–USP7 complex structure suggested that Arg309 in USP47 will cause a severe unfavorable steric hindrance with the inhibitor. In the FT671–USP7 complex structure, the Asn418 in USP7 stabilizes FT671 through a van der Waals interaction. To understand the structural basis for the observed specificity of these inhibitors, we examined the BL1 and BL2 more closely.

Upon Ub-binding, both BL1 and BL2 of USP47 move out to accommodate the C-terminal region of Ub. Both BL1 and BL2 in USP47 are well-defined in the electron density maps (Supplementary Fig. 7), and the RMSD between the two states is ~1.2 Å, significantly larger than those in each state. First, the tip of BL1 shifts by ~10 Å, and Phe300, Phe302, and Arg309 in BL1 make dramatic conformational changes upon Ub-binding. Arg309 in USP47 deserves special attention since it appears to stabilize BL1 conformation in the apo state, *i.e*., the guanidinium group of Arg309 stabilizes the switching loop by making H-bonds to His178, Asp179, Tyr175, and Tyr421 in all three molecules (Fig. 5c). The BL1 of USP7, on the other hand, is either disordered^22^ or close to that in the Ub-bound structure^30^. Second, the changes in BL2 are not as dramatic in both USP47 and USP7. However, the sequence composition is of interest. Ala416 and Ala417 of BL2 and Arg309 of BL1 in USP47 are Asn460–His461 and Asn418 in USP7. Interestingly, these three residues are highly conserved among various species (Supplementary Figs. 3 and 10).

To examine how these residues affected enzymatic activity, we mutated Arg309, Ala416, and Ala417 in USP47 to those in USP7 and vice versa. We measured the activity of these mutants using Ub-AMC with and without FT671, except for the USP7^N409R^ and USP7^N409RN460AH461A^ mutants that did not yield soluble proteins (Fig. 5d). Firstly, FT671 showed no inhibition for the wild type *c*USP47_CD_, as noted earlier^19^. Furthermore, the Arg to Asn mutation, *c*USP47_CD_^R309N^, showed no change in the activity. However, the double mutant in the BL2, *c*USP47_CD_^A416NA417H^, produced about a 40% reduction in the activity. The triple mutant, *c*USP47_CD_^R309NA416NA417H^, showed even more inhibition. FT671 showed complete inhibition for the USP7 wild type. However, *h*USP7_CD_^N460AH461A^ showed a reduction in the activity of ~80% in the presence of FT671. These results suggest that BL1 and BL2 are critical for selectivity and should be considered when designing inhibitors.

### BL3 in USP47 contributes to the catalytic activity differently from USP7

**Fig. 6:**
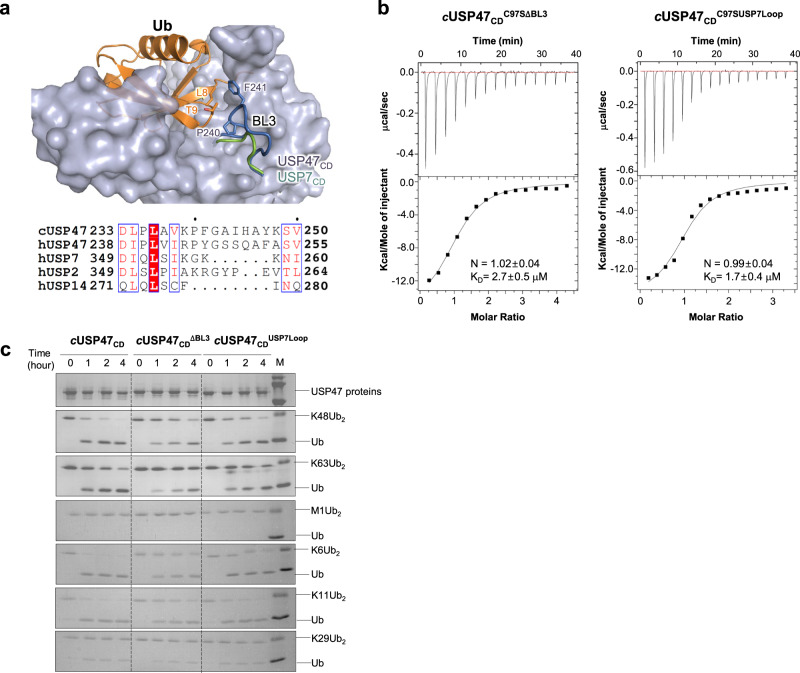
Role of the blocking loop, BL3. **a** BL3 (loop between β3 and α8) of USP47 is highlighted in blue. The corresponding loop of USP7 in the Ub complex structure (PDB code: 1NBF) is shown in green. Sequence alignment of the corresponding region in other USPs. **b** ITC data for ubiquitin-binding to (left) *c*USP47_CD_^C97SΔ236-248^ and (right) *c*USP47_CD_^C97SUSP7loop^. The error in the *K*_D_ values corresponds to the standard error of fitting a singlicate dataset. **c** Cleavage of diUb by *c*USP47_CD_, *c*USP47_CD_^ΔBL3^, and *c*USP47_CD_^USP7loop^.

In addition to the three loops mentioned, the loop between β3 and α8 (residues 236–248, referred to as BL3 hereafter) in USP47 was of interest. In the Ub-bound structure, BL3 is near the tip of the β1–β2 loop of Ub, e.g., Pro240 and Phe241 in USP47 are near Thr8 in Ub (Fig. 6a). Although BL3 is far from the catalytic Cys in the Ub-bound structure, the movement of BL3, upon Ub-binding, seems coupled to BL1 and BL2. The BL3 in USP47 is highly conserved amongst various species (Supplementary Figs. 3 and 8) and is longer than the other USPs, as seen in the figures.

To determine whether BL3 affects Ub binding or the catalytic activity, we generated two additional mutants in the Cys97Ser mutant: one with the loop deleted (ΔBL3, Δ236–248) and the other with the loop replaced by USP7 (USP7loop, ^352^SIKGK^358^). The ITC results showed the binding affinities for Ub to these mutants were about the same, or somewhat lower, than for *c*USP47_CD_^C97S^, with *K*_D_ values of 2.7 μM and 1.7 μM, respectively (Fig. 6b). Then, we tested whether the BL3 had any effect on the DUB activity of these mutants using the wild type instead of the C97S mutant. When we mutated the same loop in wild type *c*USP47_CD_ and tested against the K48- and K63-linked di-Ubs, the activities were wild type > *c*USP47_CD_^USP7loop^ > *c*USP47_CD_^ΔBL3^ for K48-linked di-Ub and wild type ≈ *c*USP47_CD_^USP7loop^ > USP47^ΔBL3^ for K63- diUb (Fig. 6c). These results suggest the BL3 in USP47 affects its DUB activity, even though their C97S mutants have a similar binding affinity for ubiquitin.

## Discussion

Here, we showed that the full-length USP47 is more active than the catalytic domain alone, while the presence of the C-terminal region enhances the DUB activity. The inactive conformation found for the catalytic domain alone, with the catalytic Cys97 ~ 10 Å away from the catalytic His420, was a result of the interactions of the hydrophobic residues (Phe167, Tyr169, and His178) with the SL region to stabilize the inactive conformation. In the Ub-bound structure, the BL1, BL2, SL, and Fingers undergo dramatic changes, with Phe300, Phe302, and Arg309 in BL1 having their sidechains adjusted to accommodate for the proper spacing of Ile35, Pro36, and Leu73 in Ub. Moreover, the Fingers region makes a roll-up motion, twisting toward the active site, to position Ub for processing. The movement of the Fingers in USP47 was greater than that in other USPs. Also, the BL3 contributes toward Ub-binding in USP47. The intrinsic flexibility of these loops and the Fingers region is attributed to the interaction between USP47 and ubiquitin.

These structural properties for USP47, including the misaligned catalytic triad, are similar to those found in the crystal structures of USP7, although Ub was covalently attached to the catalytic Cys in USP7^22^. NMR study, on the other hand, confirmed Ub-binding to USP7_CD_, but observed no chemical shifts for the residues at the active site and the surrounding loop compared to USP7_CD_ alone, i.e., the C-terminus of free ubiquitin does not enter the catalytic cleft causing the rearrangement necessary for catalysis^29^. USP7 has five UBL domains following the catalytic domain, and the presence of the last two UBL domains showed a 100-fold increase in activity compared to the catalytic domain alone^18,26,45^. Though we could not dissect the role of each UBL domain of USP47 as in USP7 due to the low sequence identity^46–48^, it is clear that the full-length USP47 is more active than either the catalytic domain alone or the catalytic domain with the first two UBL domains. Additionally, the C-terminal tail plays a role in catalysis in USP7^18,26,45–49^. In particular, two hydrophobic residues at positions 1098 and 1110 play a role in maintaining the switching loop (especially Trp285 and His294) in its active conformation. Indeed, the C-terminal of USP7 is relatively conserved amongst various species. The C-terminal of USP47 is slightly longer and not as well-conserved (Supplementary Figure 10). However, there is a highly conserved ‘Hyd-xxx-Hyd’ motif at a distal position (Leu and Ile at positions 1360 and 1362 in *h*USP47; 1316 and 1318 in *c*USP47). It is also worth noting the presence of a group of charged residues preceding the ‘Hyd-xxx-Hyd’ and an aromatic residue following the motif in both USP47 and USP7. Since they are highly conserved, they may further stabilize the SL region. USP15 also showed a misaligned active site^50^. However, the length and composition of the switching loop in USP15 differ from that of USP47 and USP7 and lacks the C-terminal tail with hydrophobic residues. The catalytic efficiency of USP4, a paralogue of USP15, was enhanced in the presence of the N-terminal DUSP-UBL domains^51^. Therefore, the distinct structural arrangement of the switching loop seems to distinguish USP7 and USP47 from the rest of the USP family.

The distinct structural features revealed for USP47 should serve as the design platform for USP47-specific inhibitors. As in USP7, the unique conformation of apo USP47 is an attractive starting point for selectivity over other USPs. Alternatively, the structural differences in BL1 and BL2, especially Arg vs. Asn and Ala-Ala vs. His-Asn, and the two Tyr residues may be the hotspot for the selective inhibitor design against the two enzymes. While His–Asn in BL2 seems important for USP7-specific inhibitors, e.g., His stabilizes the bound inhibitor and Asn locks in the conformation of BL2, in the case of USP47, two Ala residues do not seem as effective in providing this interaction. In the Ub-bound states, Arg309 in USP47 and Asn418 in USP7 interact with the Ub-tail. However, in the apo states, Asn418 in USP7 is located on the surface, while Arg309 in USP47 stabilizes the SL and blocks the entrance to the binding pocket, which relates to a movement of about 10 Å for the BL1 in the case of USP47. The flexibility seen for the BL1 in USP7 may be because the adjacent BL3 in USP7 is shorter since the conformational changes in these loops seem to be concerted. The structural differences between USP47 and USP7 revealed in BL1, BL2, and BL3 should be a starting point for designing specific inhibitors.

USP7 has been studied widely as a target for antitumor therapies, whereby USP7 regulates the MDM2–p53 pathway. In fact, USP7 was identified initially as a DUB for p53, although it was later discovered that MDM2 is a more efficient substrate of USP7 that modulates p53 stability. Owing to the importance of p53 regulation in cancer, USP7 has been studied extensively from the early stages of DUB research. Conversely, USP47 is phylogenetically similar to USP7, yet studies on its function and mechanism have been conducted only over the last ten years. Previously, we have reported that USP47 is also involved in the MDM2–p53 pathway^9^. While USP7 stabilizes MDM2 directly, USP47 regulates MDM2 by deubiquitinating RPS2, suggesting that USP47 could be a delicate regulator of p53 in some stress responses. Consistent with previous reports, we observed that depletion of USP47 reduced cell proliferation only in the p53-positive A549 cell line and not in H1299 cells, which lack p53 expression. These data correlate well with previous findings showing that USP47 regulates the MDM2–p53 pathway.

In addition to regulating the MDM2–p53 pathway, USP7 and USP47 have many redundant roles. Both regulate various signaling pathways, such as the inflammasome pathway^4^, WNT signaling^52,53^, Hedgehog signaling^5,54^, etc. USP7 and USP47 deubiquitinate NLRP3 and control the assembly and activation of inflammasomes in macrophages^4^. USP7 functions as a WNT activator through the deubiquitination of β-catenin and promotes the growth of APC-mutated colon cancer cells^53^. USP47 also deubiquitinates β-catenin, which is crucial for tumorigenesis and animal development^52^. USP7 and USP47 regulate Hedgehog signaling by deubiquitinating Ci/Gli and β-TrCP, respectively^5,52^. USP7 and USP47 share substrates such as the Yes-associated protein (YAP), which functions as the main effector of the Hippo pathway^55,56^.

Some biological functions appear to be redundant between USP7 and USP47. However, the role of each enzyme is not completely taken over by the other because the modulators that control each USP7 and USP47 signaling pathway are different. Our results, which showed that the depletion of both USP7 and USP47 could more potently inhibit cell proliferation relative to the individual depletion of either alone, also support. While USP47 and USP7 both regulate the MDM2–p53 pathway, USP7 stabilizes MDM2 directly, while USP47 regulates MDM2 by deubiquitinating RPS2^9^. Both DUBs bind to β-TrCP and reverse substrate ubiquitination by β-TrCP^5,54^. However, USP47 can both interact with and deubiquitinate β-TrCP. The interaction of USP47 and β-TrCP is regulated by ERAP1^5^, suggesting that each has a fine-tuned regulatory mechanism. Also, SATB1^6^, DNA polymerase β^27^, etc., are deubiquitinated only by USP47. These differences may have other implications for cancer resistance, as in a recent report where USP47 can overcome tyrosine kinase inhibitor resistance in BCR-ABL^T315I^-induced chronic myelogenous leukemia^10^. Furthermore, Yang *et al*. recently proposed targeting USP47 with a small molecule inhibitor as a novel potential therapy for diffuse large B-cell lymphoma and other hematologic malignancies characterized by mutant EZH2 expression^11^.

Several highly selective inhibitors, including ones developed based on the co-crystal structures of inhibitors bound to USPs, have been reported recently^10,57–60^. In particular, compound 41, both highly potent and selective against USP7, is orally bioavailable and demonstrates tumor growth inhibition in both p53 wild type and p53 mutant cancer cell lines in xenograft studies^61^, suggesting that the inhibition of USP7 could suppress tumor growth through multiple pathways, thereby delineating a possible clinical application. Dual inhibitors of USP7 and USP47 have been used in various studies. However, the development of targeted inhibitors against USP47 has been limited thus far. We hope that the findings in this study serve as a starting point for drug discovery against USP47, either alone or with USP7.

## Methods

### Cell culture

All the cell lines used in this study were purchased from Korea Cell Line Bank (KCLB). HEK293T cells were cultured in DMEM and p53-positive HCT116 (p53+/+) and p53-deficient HCT116 (p53+/+) cells were cultured in RPMI-1640 medium containing 10% fetal bovine serum and 1% penicillin and streptomycin. The cells were maintained in a humidified incubator with 5% CO_2_ at 37 °C. All the cell lines used in this study have been authenticated by KCLB and confirmed to be free of mycoplasma contamination prior to use.

### Transfection and siRNAs

For transient transfection, HEK293T cells were transfected with plasmids using 2 M CaCl_2_ and 2X HBS buffer (50 mM HEPES, 10 mM KCl, 12 mM glucose, 280 mM NaCl, 1.5 mM Na_2_HPO_4_, pH 7.05) for 24 hr, and HCT116 cells were transfected with siRNAs using LipofectamineTM 3000 (Invitrogen) for 48 hr, following the manufacturer’s instructions. siRNA sequences were as follows: USP7: #1; (purchased from Santa Cruz Biotechnology (#sc-41521)) and #2; 5′-ACCCUUGGACAAUAUUCCU-3′, USP47: #1; 5′-AAGCTACTCCTACTCATCTATTT-3′ and #2; 5′-TGAAAAGGGATGTGCAAAA-3′. CONi was used as a negative control and was purchased from Bioneer.

### Western blot analysis

A total of 10–50 μg protein was extracted from cells and used for Western blot analysis. Briefly, the cells were lysed using protein lysis buffer (50 mM Tris–Cl, 150 mM NaCl, 1% Triton X-100, 1 mM EDTA, 200 mM Na3VO4, 1x proteinase inhibitor, pH 7.4) and the protein concentration was measured using a Micro BCA^TM^ Protein Assay kit (Thermo Scientific), based on the standard curve of BSA. The antibodies used for immunoblot analysis were purchased and assessed for equal loading as follows: rabbit anti-USP7 (#A300-034, dilution ratio 1:1000) and rabbit anti-USP47 (#A301-048A, 1:1000) from Bethyl Laboratories; mouse anti-Flag (#F1804, 1:1000) from Sigma Aldrich; rabbit anti-ubiquitin (#3933, 1:2000) from Cell Signaling; mouse anti-p53 (#sc-126, 1:1000), and mouse anti-HSP90α/β (#sc-13119, 1:5000) from Santa Cruz Biotechnology; rabbit anti-β-actin (#LF-PA0207, 1:5000) from AbFrontier. Samples were analyzed using SDS-PAGE, and chemiluminescence was measured using the Ez-Capture MG imaging system (ATTO Corporation).

### Cell proliferation assay (WST-1 assay)

USP47 siRNA (5′-TGAAAAGGGATGTGCAAAA-3′) was synthesized (Genolution), while USP7 siRNA was purchased (Santa Cruz Biotechnology, #sc-41521). HCT116 (p53 + /+) and HCT116 (p53-/-) cells were seeded in 96-well plates at a density of 5000 cells per well, and then, transfected with siRNA targeting USP7 or USP47, either alone or together. After transfection, a cell proliferation assay was conducted each day using EZ-Cytox (Daeilbio, WST-1; #EZ-1000), using the manufacturer’s instructions. For P50429, the cells were seeded at 3000 or 6000 cells per well with an inhibitor concentration of 25 μM and 12.5 to 100 μM, respectively. EZ-Cytox (DoGEN, EZ-3000) was diluted using media and incubated for 30 min. The absorbance was measured at a wavelength of 450 nm using a microplate absorbance reader (Bio-Rad).

### Colony formation assay

HCT116 (p53+/+) and HCT116 (p53−/−) cells were transfected with siRNA targeting USP7 or USP47, either alone or together. The cells were re-suspended with trypsin-EDTA, and 1000 cells were seeded in 35 mm dishes 48 h after transfection. After 10 days, the cells were washed with PBS and fixed in 4% paraformaldehyde for 10 min before being stained with Crystal violet for 30 min and washed with PBS.

### Immunoprecipitation and DUB assay

HEK293T cells transfected with pFlag-CMV-2 vector, pFlag-USP7, or pFlag-USP47 were treated with drugs, as indicated, and lysed by protein lysis buffer (20 mM Tris–Cl, 150 mM NaCl, 1 mM EDTA, 1 mM EGTA, 1% Triton X-100, 2.5 mM sodium pyrophosphate, 50 mM NaF, 5 mM β-glycerophosphate, 1 mM Na_3_VO_4_, 1x protease inhibitor, pH 7.5). Cell lysates of about 2 mg were incubated with EZview^TM^ Red anti-Flag M2 affinity gel (Sigma Aldrich) for 4 hr at 4 °C. Flag affinity gel was washed 5 times with lysis buffer and the supernatant was removed carefully with a syringe after the last wash step. For elution, Flag agarose was incubated with 3× Flag peptide (Sigma Aldrich) in elution buffer (15 μg of Flag peptide in 50 μl of lysis buffer) for 30 min at 4 °C. The supernatants were collected as immunoprecipitates. The DUB activities of USPs were tested using recombinant K48-linked ubiquitin chains (UB2-7, Boston Biochem #UCH-230). The immunoprecipitates were incubated with recombinant K48-linked ubiquitin chains for 30 min at 30 °C, and the DUB reaction was stopped by adding 6x SDS sample buffer and boiling for 10 min at 95 °C. Samples were analyzed by Western blot and detected with the anti-ubiquitin antibody.

### Enzymatic activity and inhibition assays

Ubiquitin chain cleavage assays were performed with both K48- and K63-linked di-Ub chains at 37 °C. Enzymes and substrates were diluted in the reaction buffer (20 mM HEPES, pH 7.4, 150 mM NaCl, and 5 mM DTT) to a concentration of 5 μM for the substrate and 0.5 μM for the enzymes. At the indicated time points following the reaction initiation, 10 μL reaction aliquots were taken and the reaction was stopped by adding SDS sampling buffer and analyzed using SDS-PAGE. Then, the 4–15% gradient gel was stained with Coomassie Brilliant Blue R-250. USP enzymatic activity and inhibition assays were measured at room temperature using the cleavage-sensitive fluorogenic substrate Ub-AMC (Boston Biochem, U-550). The deubiquitination assay was performed following the protocol by Ernst et al.^30^. Briefly, enzyme activity assays were performed in assay buffer in 20 mM HEPES (pH 7.4), 100 mM NaCl, and 5 mM DTT) containing *c*USP47_CD_ (in 50 nM), *h*USP47_CD-UBL12_ (in 100 nM) or *h*USP7_CD_ (in 100 nM) constructs and serial dilutions of Ub-AMC (final 35 μM). The AMC fluorescence emission was monitored at 460 nm (excitation at 380 nm) for 30–60 min using a SpectraMax M3 plate reader (Molecular Devices). The steady-state enzyme kinetic parameters were obtained by fitting the initial velocity data to the Michaelis–Menten equation (*V*_0_ = *V_max_*[S]/(*K*_M_ + [S]) using GraphPad Prism 5 (GraphPad Inc.). The *k*_cat_ values are from the equation *k*_cat_ = *V*_max_/[E]o, where [E]o is the total enzyme concentration. For IC_50_, USP47 (in 50 nM) or USP7 (in 100 nM) were incubated with varying concentrations (0–50 μM) of P50429 inhibitor in the assay buffer, adding 2% DMSO for 10 min at 25 °C. For the inhibition assay using Ub-AMC (in 35 μM), USP47 (in 50 nM) or USP7 (in 100 nM) were incubated with concentrations of FT671 (in 5 μM) in the assay buffer with 2% DMSO for 10 min at 25 °C. Residual enzymatic activity was determined by measuring the rate of hydrolysis ubiquitin-AMC by USP47 and USP7 in triplicate. The data were fitted using GraphPad Prism 7 (GraphPad Inc.). Fluorescence intensity was recorded every 45 s for 90 min. Data are presented as the mean ± SD (*n* = 3).

### Protein expression and purification

A DNA fragment encoding the catalytic domain of the *c*USP47 gene from *Caenorhabditis elegans* (Swiss Prot entry: Q22240, residues 1–508) and the catalytic domain of the *h*USP7 gene from humans (Swiss Prot entry: Q93009, residues 208–560) were amplified and cloned into pET-28a (Novagen) with a His_6_-tag at the N-terminal. All primers used in this study are in Supplementary Table 1. Human USP47 (Swiss Prot entry Q96K76) encodes 1287 residues. Constructions of *h*USP47_CD-UBL12_ (residues 1–794) were amplified and cloned into the pET-32a thioredoxin-His_6_-tag at the N-terminal. Various mutants of *c*USP47_CD_ (C97S, C97A, F167A, W169A, H178F, H178A, C97SF167A, C97SW169A, C97S/H178F, C97SH178A, R308N, A416NA417H, and R308NA416NA417H) and *h*USP7_CD_ (C223S, F283A, W285A, H294F, H294A, and N460AH461A) were generated using site-directed mutagenesis (Stratagene) and were cloned into the pET-28a vector, similar to the wild type. The deletion (USP47_CD_^ΔBL3^) mutant and *c*USP47_CD_^USP7loop^ constructs (^236^LAVKPFGAIHAY^248^) were replaced by a short loop, based on USP7 (PDB code 1NBF) residues 352–358 (^352^SIKGK^358^), and cloned into the pET-28a vector, respectively. The mutations were verified by DNA sequencing. All constructs were expressed in *E. coli* BL21 (DE3) codon plus RIL (Agilent) strain.

All proteins were expressed by induction with 0.1 mM IPTG at 18 °C overnight. Cells were harvested by centrifugation and re-suspended in buffer containing 20 mM HEPES (pH 7.2), 150 mM NaCl, 2 mM β-mercaptoethanol, and 1 m*M* phenylmethylsulfonyl fluoride, and were disrupted by sonication, and the crude lysate was centrifuged at 18,000 rpm (Hanil Supra 22 K) for 40 min at 4 °C, and the cell debris was discarded. The supernatant was loaded onto a nickel-chelated Hi-trap column (GE Healthcare) and eluted with a linear gradient of 25–500 mM imidazole in 20 mM HEPES (pH 7.2), 150 mM NaCl, and 2 mM β-mercaptoethanol. The pooled fraction was further purified by gel filtration chromatography on HiLoad 26/60 Superdex-75 (GE Healthcare) and HiLoad 26/60 Superdex-200 (GE Healthcare) pre-equilibrated with 20 mM HEPES (pH 7.2), 150 mM NaCl, and 2 mM DTT. All mutants of USP47 and USP7 were purified in the same way as the wild type protein, and the purity of the proteins at each stage was checked using 15% SDS-PAGE. The purified USP47 constructs were concentrated to 20 mg/mL using a VIVASPIN20 (Sartorius) concentrator and stored at −80 °C until use. The selenomethionine (Se-Met) substituted USP47 was produced in *E. coli* BL21-codon Plus RIL-X (DE3) methionine auxotroph (Agilent) and purified following the same procedure described for the native protein.

### Crystallization

Diffraction-quality crystals were obtained by mixing the protein in 20 mM HEPES (pH 7.2), 150 mM NaCl, and 2 mM DTT with a reservoir solution containing 0.2 M imidazole-malate (pH 5.5) and 18% (*v*/*v*) PEG 4000 for 2 days. They were cryo-protected using the reservoir solution supplemented with 20% additional glycerol and were flash-frozen in liquid nitrogen. To obtain the *c*USP47_CD_^C97S^:Ub complex, purified cUSP47 in 20 mM HEPES (pH 7.2), 150 mM NaCl, and 2 mM DTT was incubated with Ub at a molar ratio of 1:2 at 4 °C for 2 hr prior to the crystallization attempts. *c*USP47_CD_^C97S^:Ub complex crystals were obtained by mixing an equal volume of protein solution and reservoir solution (100 mM bis–Tris (pH 5.5), 25% PEG 3350, and 50 mM MgCl_2_).

### Diffraction data collection and processing

Diffraction data were collected at −173 °C using the beamline BL-5C equipped with an ADSC Quantum 315r CCD detector of Pohang Light Source (Pohang, Korea). The Se-Met labeled crystals were obtained from the identical condition as the native crystals and diffracted to 3.2 Å resolution. All datasets were processed and scaled using the program DENZO and SCALEPACK from the HKL2000 program suite^62^.

### Structure determination and refinement

The structure of the *c*USP47 catalytic domain was solved using single anomalous dispersion (SAD) with a mean FOM of 0.39 to a resolution of 3.2 Å. Four potential Se sites were identified using SOLVE^63^, while density modification and automated model building were conducted using RESOLVE^63^, and further improvement was made by Phaser^64^. The iterative manual building was performed using COOT^65^ and refinement was performed using PHENIX^66^. A few regions that were not well defined in the electron density maps, *e.g*., 1–77, 119–132, 329–381 in all three molecules; 459–462 in molecules B and C; 263–379 in molecule C, were omitted from the final model. The complex was solved by molecular replacement using a molecule of USP47 and ubiquitin (PDB code 1UBQ) as a search model. After rigid-body refinement, model building, and refinement were iteratively performed using COOT and PHENIX.

### Structure analysis

The final models were validated using PROCHECK^67^. Solvent accessible area and interaction area were calculated by PISA (http://www.ebi.ac.uk/msd-srv/prot_int/pistart.html). The sequence alignment was performed using ClustalW and the image was produced using ESPript 3.0 (http://espript.ibcp.fr). The figures were generated using PyMOL (http://www.pymol.org). Statistics on data collection and refinement are provided in Table 1.

### Isothermal titration calorimetry (ITC)

Ubiquitin-binding to USP47 was performed using the ITC200 instrument (GE Healthcare) at 25 °C, and the data were analyzed using the program ORIGIN 7.0. Prior to titration, the protein samples were centrifuged at 18,188×*g* at 4 °C for 5 min to remove any debris. Experiments were conducted by titrating 500 µM (except for the mutants of *c*USP47_CD_^H178F^ and *c*USP47_CD_^H178A^ using 1 mM) monoubiquitin into the cell containing ~25–30 µM of prepared wild type and each mutant of USP47 protein in identical buffer with injectant. All proteins were prepared by dialyzing in a buffer of 20 mM HEPES (pH 7.4), and 100 mM NaCl at 25 °C. The injections were 2 μL each, with an injection interval of 150 s. The stoichiometry (n), association constant (*K*_D_), and the change in enthalpy (Δ*H*), were obtained by using a nonlinear least-squares curve-fitting algorithm.

### Statistics and reproducibility

Results are shown as mean ± SD of at least three independent experiments unless otherwise indicated in the figure legends. The comparison of different groups was carried out using a two-tailed unpaired Student’s *t*-test, and the *P*-value < 0.05 was considered statistically significant and reported as in legends.

### Reporting summary

Further information on research design is available in the Nature Portfolio Reporting Summary linked to this article.

### Supplementary information

Supplementary Information

Description of Additional Supplementary Files

Supplementary Data 1

Reporting Summary

## Data Availability

The crystal structures have been deposited in the Protein Data Bank with accession codes 8ITN and 8ITP for *c*USP47CD and *c*USP47CDC97S:Ub, respectively. They are available at the http://www.rcsb.org. The Alpha Fold model for human USP47 is available as Q96K76 at http://www.Alphafold.ebi.ac.uk. Uncropped gels are in Supplementary Figure 11, and source data are in Supplementary Data 1. Further information and requests for resources and reagents should be directed to Eunice E. Kim (eunice@kist.re.kr) or Eun Joo Song (esong@ewha.ac.kr).
