# Supplementary material for: Structural and functional characterization of USP47 reveals a hot spot for inhibitor design
Source: Commun Biol. 2023 Sep 22;6:970. doi: 10.1038/s42003-023-05345-5 (PMC10516900; doi:10.1038/s42003-023-05345-5)
Supplement: Supplementary file 1 — Supplementary Information [file 42003_2023_5345_MOESM1_ESM.pdf]

Supplementary Information

Structural and functional characterization of USP47 reveals a hot spot for inhibitor design

Sang Chul Shin[1, †], Jinyoung Park[1,2], Kyung Hee Kim[1], Jung Min Yoon[1,3], Jinhong Cho[1], Byung Hak Ha[1, ‡], Yeonji Oh[4], Hyunah Choo[2,4], Eun Joo Song[3,*], and Eunice EunKyeong Kim[1,*]

Supplementary Table 1. Primers used in the study.

Supplementary Figure 1. Transfection efficiency of siRNA targeting USP47 or USP7.
Supplementary Figure 2. SDS-PAGE for USP47 and USP7 used for enzymatic analysis.
Supplementary Figure 3. Sequence alignment of $USP47_{CD}$ and $USP7_{CD}$.
Supplementary Figure 4. ITC analysis of Ub-binding to *c*$USP47_{CD}$.
Supplementary Figure 5. Electron density map at the active site of *c*$USP47_{CD}^{C97S}$:Ub.
Supplementary Figure 6. Comparison of crystal structures *c*$USP47_{CD}$ and model for *h*$USP47_{CD}$.
Supplementary Figure 7. Structural comparison of BL1, BL2, and BL3 of $USP47_{CD}$.
Supplementary Figure 8. Sequence alignment of the catalytic domain of USP47 with other USPs.
Supplementary Figure 9. Superposition of the apo *c*USP47 structure onto the FT671-bound USP7.
Supplementary Figure 10. Sequence alignment of the C-terminal region of USP47s and USP7.
Supplementary Figure 11. Uncropped membranes used in this study.

Supplementary Table 1. Primers used in the study.

	Primer Name	5` --> 3`
1	cUSP47_1_BamH1_Forward	CACCGGATCCATGGTACGGGTCGAGGACTCGAACGGC
2	cUSP47_508_Xho1_Reverse	CCGCTCGAGTTATTCCGCCTCTCGTTCAAGTC
3	hUSP47_1_Nde1_Forward	GGGAATTCCATATGGTGCCCGGCGAGGAGAACCAACTGGTC
4	hUSP47_794_Xho1_Reverse	CCGCTCGAGTCATTTGAGTTTTTCAGTGCT
5	hUSP47_1_Not1_Forward	AAGGAAAAAAGCGGCCGCGGTGCCCGGCGAGGAGAACCAACTG
6	hUSP47_477_Kpn1_Reverse	CGGGGTACCTCAATCCTTCAGTCTATAGATCAGCATATATGC
7	hUSP47_794_Kpn1_Reverse	CGGGGTACCTCATTTGAGTTTTTCAGTGCT
8	hUSP47_1287_Kpn1_Reverse	CGGGGTACCTCAGTCTTGAGTCAGATCTTTATTTGGTGCTCCATCCA
9	cUSP47_C97S_Forward	ACCCAGGCAATGACTGCATATTTGAACAGTCTT
10	cUSP47_C97S_Reverse	AAGACTGTTCAAATATGCAGTCATTGCCTGGTT
11	cUSP47_C97A_Forward	ACCCAGGCAATGACTGCATATTTGAACAGTCTT
12	cUSP47_C97A_Reverse	AAGACTGTTCAAATATGCAGTCATTGCCTGGTT
13	cUSP47_F167A_Forward	AAAGATCTAACGCAAAGTGCAGGATGGACATCG
14	cUSP47_F167A_Reverse	ATTCGATGTCCATCCTGCACTTTGCGTTAGATC
15	cUSP47_W169A_Forward	ACGCAAAGTTTTGGAGCAACATCGAATGAAGCA
16	cUSP47_W169A_Reverse	GTATGCTTCATTCGATGTTGCTCCAAAACTTTGCGT
17	cUSP47_H178A_Forward	GAAGCATACGATCAGGCAGATGTTCAGGAA
18	cUSP47_H178A_Reverse	AAGTTCCTGAACATCTGCCTGATCGTATGC
19	cUSP47_H178F_Forward	GAAGCATACGATCAGTTCGATGTTCAGGAA
20	cUSP47_H178F_Reverse	AAGTTCCTGAACATCGAACTGATCGTATGC
21	cUSP47_R309N_Forward	AATACAATGCATAATATTAAATTAAAC
22	cUSP47_R309N_Reverse	GTCGTTTAATTTAATATTATGCATTGT
23	cUSP47_A416NA417H_Forward	CATTCTGGAAATAATCATGGGGGACAT
24	cUSP47_A416NA417H_Reverse	GTAATGTCCCCCATGATTATTTCCAGA
25	cUSP47_ΔBL3(236-248 a.a)_Forward	TACTTTCTTGATCTACCGTTAAAAAGTGTCGAAGAAGCGTTG
26	cUSP47_ΔBL3(236-248 a.a)_Reverse	CAACGCTTCTTCGACACTTTTTAACGGTAGATCAAGAAAGTA
27	cUSP47_USP7loop_Forward	TTTCTTGATCTACCGTTAAGCATCAAGGGCAAGAAAAGTGTCGAAGAAGCG
28	cUSP47_USP7loop_Reverse	CGCTTCTTCGACACTTTTCTTGCCCTTGATGCTTAACGGTAGATCAAGAAA
29	hUSP7_208_BamH1_Forward	CACCGGATCCAAGAAGCACACAGGCTACGTC
30	hUSP7_560_Xho1_Reverse	CCGCTCGAGCTATTCCTGCCGCTCCTTCCGCT
31	hUSP7_F283A_Forward	AAGTTAACAAAGTCAGCAGGGTGGGAAACT
32	hUSP7_F283A_Reverse	ATCTAAAGTTTCCCACCCTGCTGACTTTGTTAA
33	hUSP7_W285A_Forward	ACAAAGTCATTTGGGGCAGAAACTTTAGAT
34	hUSP7_W285A_Reverse	GCTATCTAAAGTTTCTGCCCCAAATGACTT
35	hUSP7_H294A_Forward	TTAGATAGCTTCATGCAAGCAGATGTTCAGGAG
36	hUSP7_H294A_Reverse	AAGCTCCTGAACATCTGCTTGCATGAAGCTATC
37	hUSP7_H294F_Forward	TTAGATAGCTTCATGCAATTCGATGTTCAGGAG
38	hUSP7_H294F_Reverse	AAGCTCCTGAACATCGAATTGCATGAAGCTATC
39	hUSP7_N460AH461A_Forward	AGTGGAGATGCTGCTGGTGGACATTAT
40	hUSP7_N460AH461A_Reverse	CACATAATGTCCACCTGCTGCATCTCCACT
41	hUB_1_Nde1_Forward	GGAATTCCATATGATGCAGATCTTCGTGAAGACTCTG
42	hUB_76_Sal1_Reverse	CCACGCGTCGACTCACCCACCTCTGAGACGGAGCACCAGGTGCAG

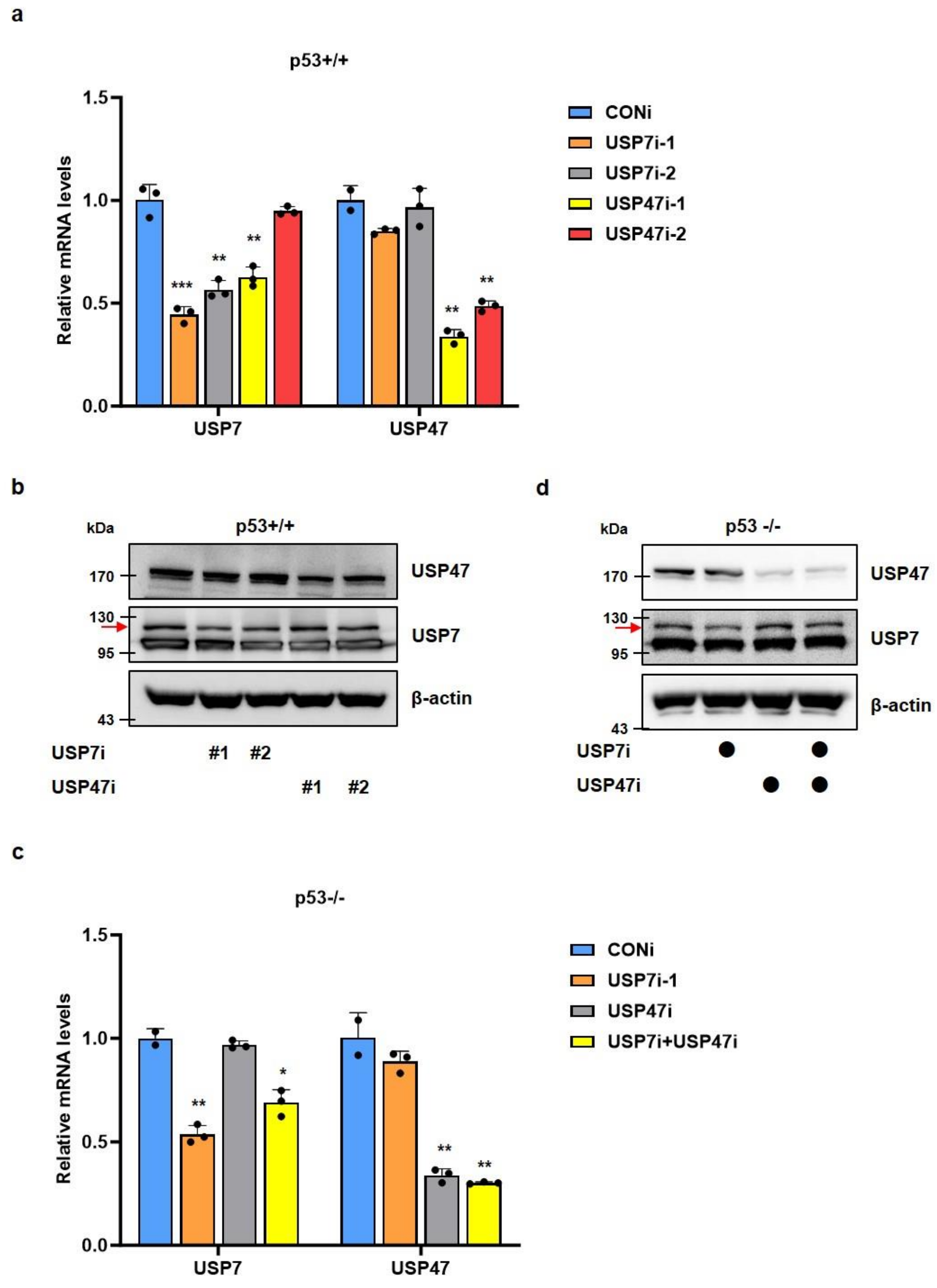

Supplementary Figure 1. Transfection efficiency of siRNA targeting USP47 or USP7.

(**a**, **b**) HCT116 (p53+/+) cells were transfected with two siRNA targeting USP7 or USP47, respectively. (**a**) Q-PCR analysis was conducted to determine the relative expression levels of USP7 and USP47. (**b**) Western blot analysis was performed to detect USP7 and USP47 protein levels. (**c, d**) HCT116 (p53-/-) cells were transfected with siRNA targeting USP7 or USP47 alone or together. (**c**) Q-PCR analysis was conducted to determine the relative expression levels of USP7 and USP47. (**d**) Western blot analysis was performed to detect USP7 and USP47 protein levels. The data represent the mean ± SD (*;p<0.05, **;p<0.005, ***;p<0.0005).

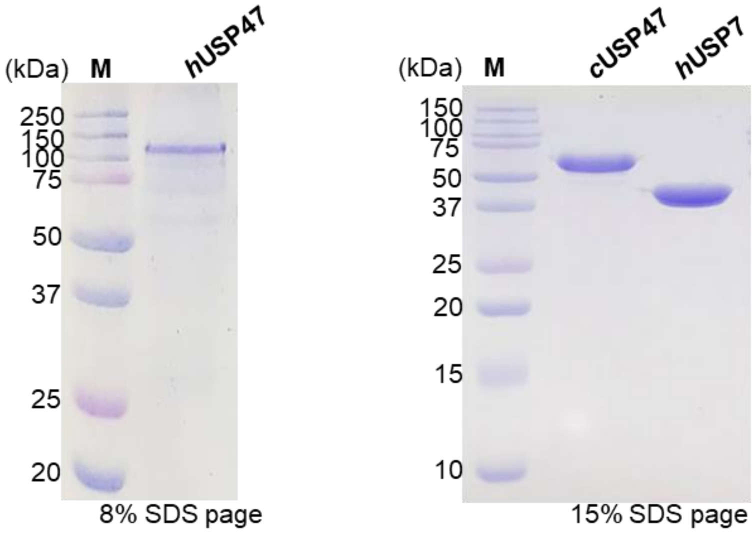

Supplementary Figure 2. SDS-PAGE for USP47 and USP7 used for enzymatic analysis.

Final purification product of *h*USP47$_{CD\text{-}UBL12}$ (residues 1-794), *c*USP47$_{CD}$ (residues 1-508), and *h*USP7$_{CD}$ (residues 208-560) shown on SDS-PAGE gel (4 μM). Results are visualized using SDS-PAGE and Coomassie blue staining.

a

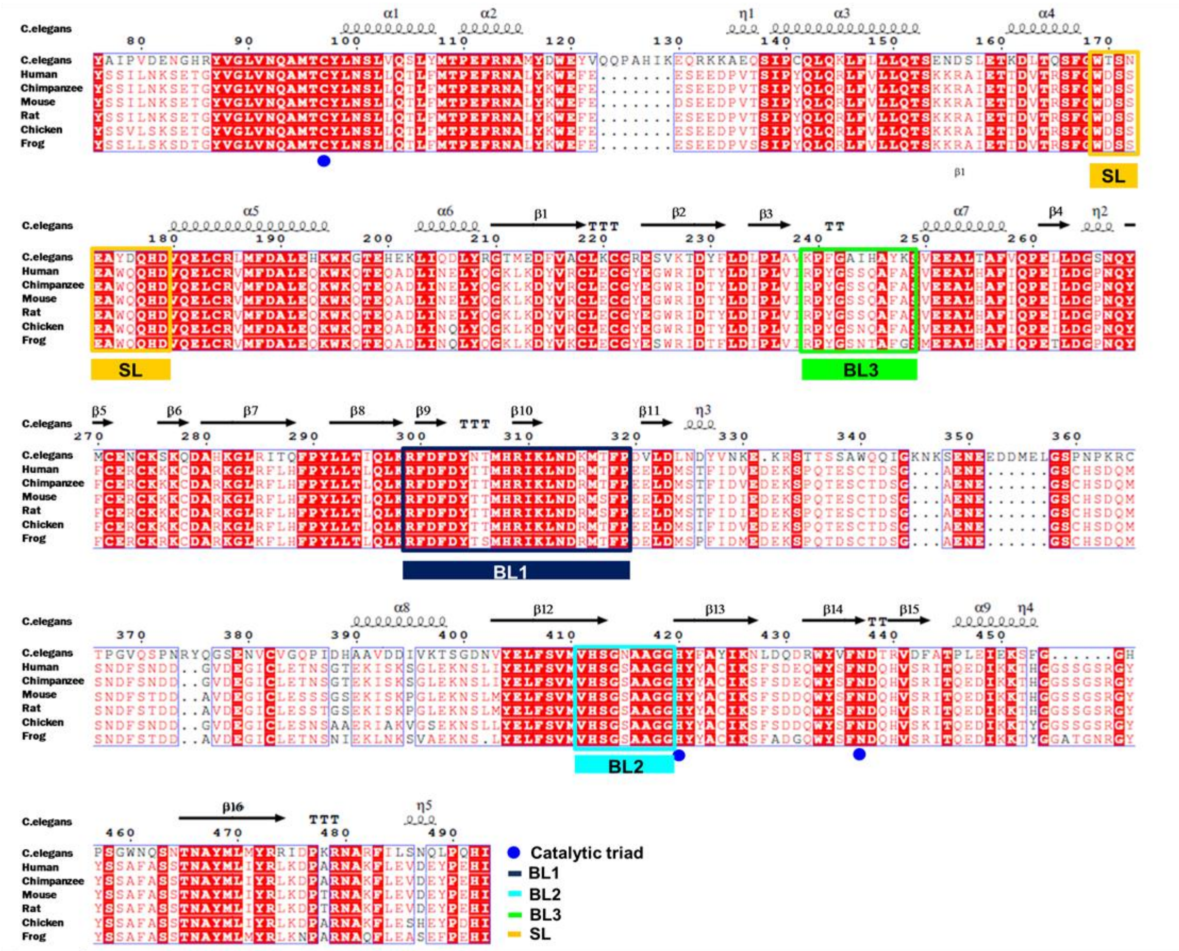

C.elegans
Human
Chimpanzee
Mouse
Rat
Chicken
Frog
SL
BL1
BL2
BL3
Catalytic triad

b

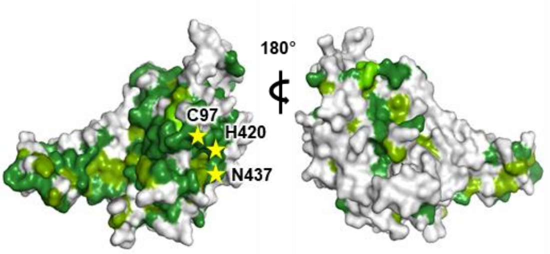

180°
C97
H420
N437

c

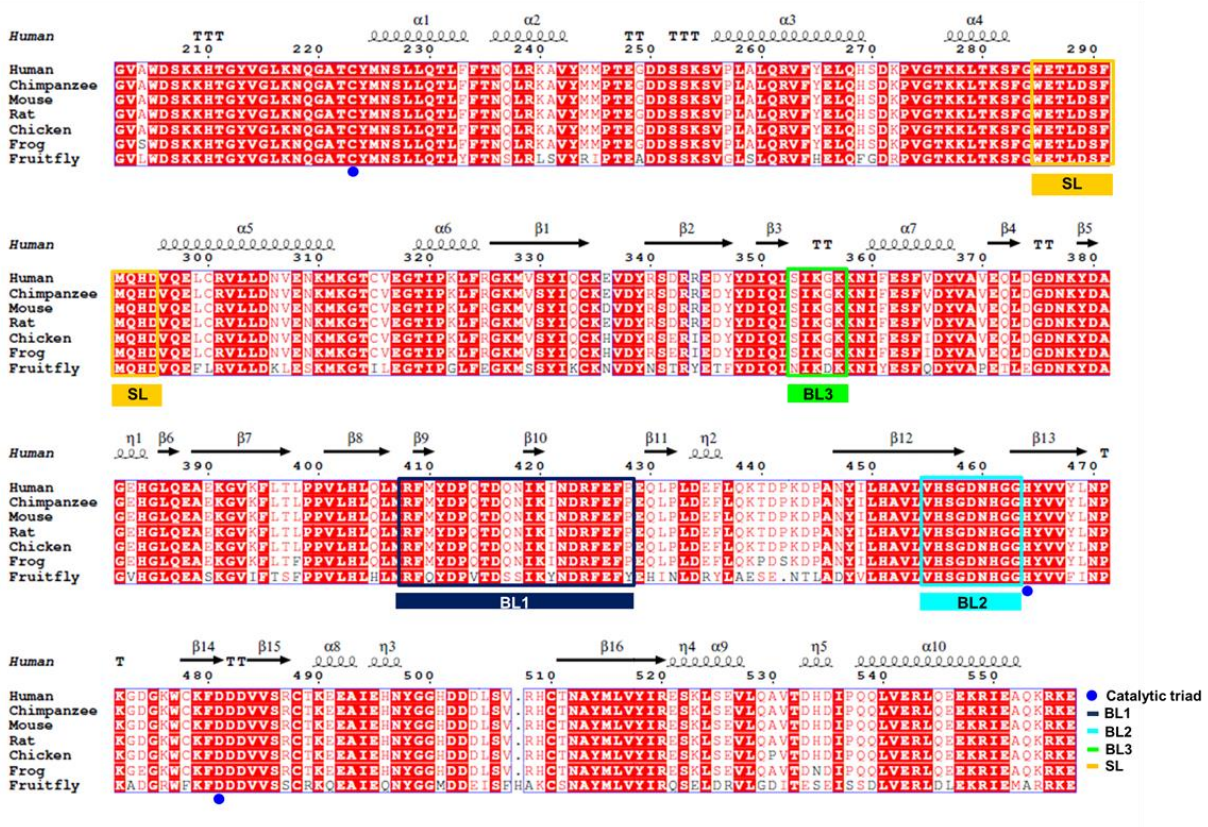

Supplementary Figure 3. Sequence alignment of USP47 and USP7 catalytic domains.

(a) Sequence alignment of the USP47 catalytic domain. The sequence of USP47s from *C.elegans* (Q22240), human (Q96K76), chimpanzee (H2RDB9), mouse (Q8BY87), rat (A0A0G2JUX4), chicken (E1C1R4), and African clawed frog (Q5U252). The entry codes in the Swiss Prot database are given in (). The catalytic residues (blue dot), BL1 (purple), BL2 (cyan), BL3 (green), and SL (yellow) are highlighted. Within each alignment, strictly conserved residues are written in white and highlighted in red. Residues with >70% conservation are surrounded by a blue box and written in red. **(b)** Molecular surface presentation of USP47 based on sequence conservation, colored gray to dark green as the degree of conservation increases. The catalytic residues are indicated by the yellow stars. **(c)** Sequence alignment of the USP7 catalytic domain. The sequence of USP7s from human (Q93009), chimpanzee (A0A2I3SG21), mouse (Q6A4J8), rat (Q4VSI4), chicken (Q6U7I1), African clawed frog (A0A8J0TNB1), and fruit fly (Q9VYQ8). The entry codes in the Swiss Prot database are given in (). The catalytic residues, BL1, BL2, BL3, and SL are highlighted in the same color scheme as in USP47. ClustalX.3.0 and ESPript 3.0 (http://www.espript.ibcp.fr) were used for alignment and image, respectively.

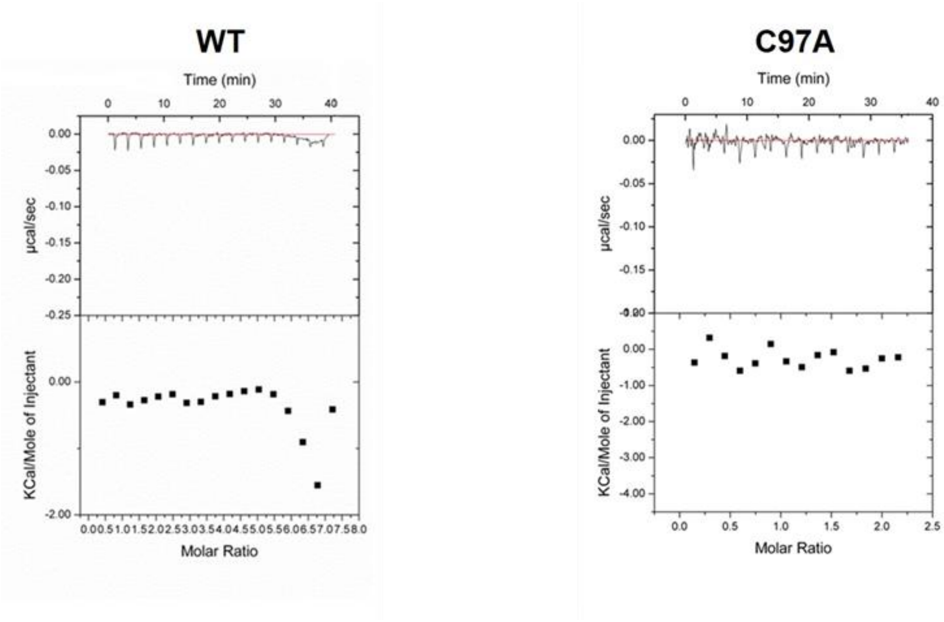

Supplementary Figure 4. ITC analysis of Ub-binding to *c*USP47 catalytic domain.

ITC binding curve for Ub to the catalytic domain of wild type and *c*USP47$_{CD}^{C97A}$ mutant. The lower panel shows the integrated heat data against the molar ratio of Ub to *c*USP47, respectively.

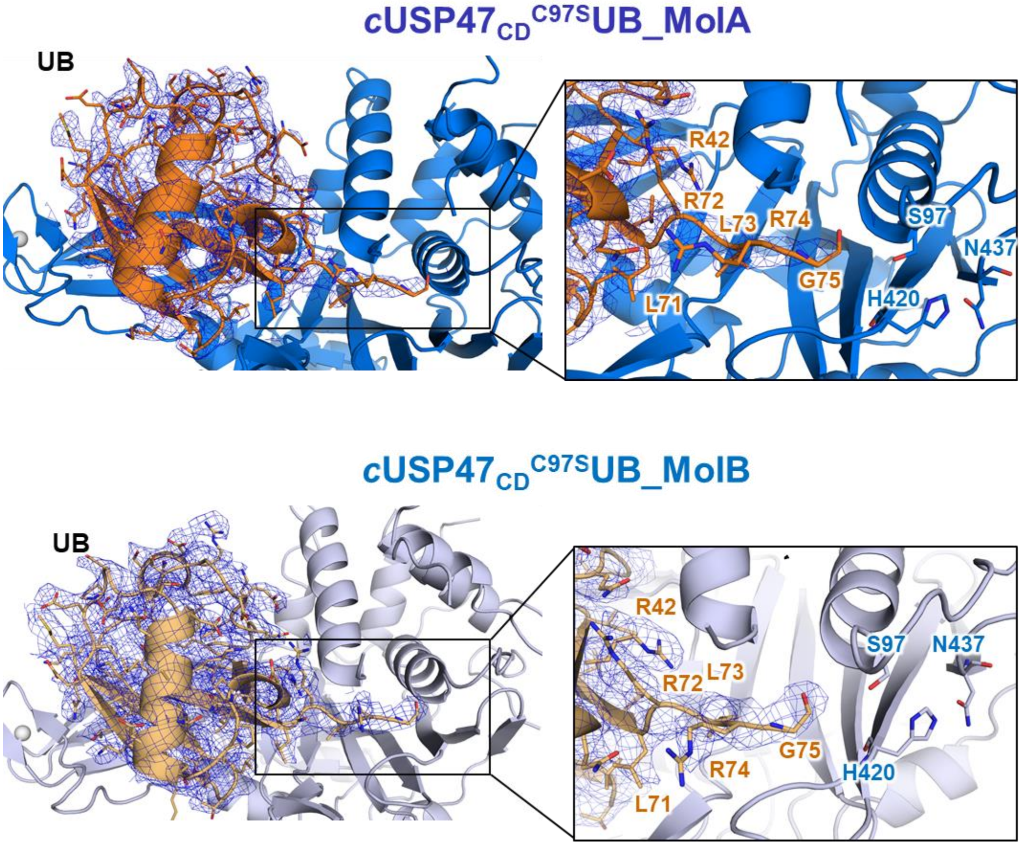

Supplementary Figure 5. Electron density map at the active site of *c*USP47$_{CD}^{C97S}$:Ub.

Electron density map of Ub in the *c*USP47$_{CD}^{C97S}$:Ub structure with ribbon representation of USP47 shown in blue and ubiquitin in orange in molecule A and light blue and light orange in molecule B. The 2*F*o-*F*c electron density map is contoured at 1σ level. The close-up view shows the C-terminal tail of ubiquitin with the catalytic triad of *c*USP47$_{CD}^{C97S}$.

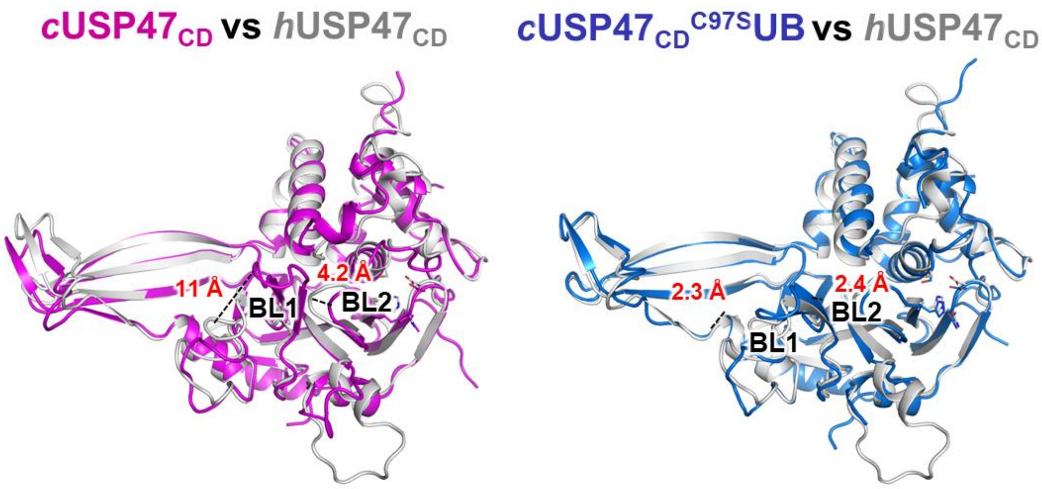

Supplementary Figure 6. Comparison of crystal structures *c*USP47$_{CD}$ and model for *h*USP47$_{CD}$.

The *h*USP47$_{CD}$ model (AF-Q96K76-F1) from AlphaFold Protein Structure Database in gray is superposed onto the free and Ub-bound *c*USP47$_{CD}$ crystal structures shown in magenta and blue, respectively. The RMSD values are 1.51 and 0.82 Å, respectively, with the human model being closer to the Ub-bound structure.

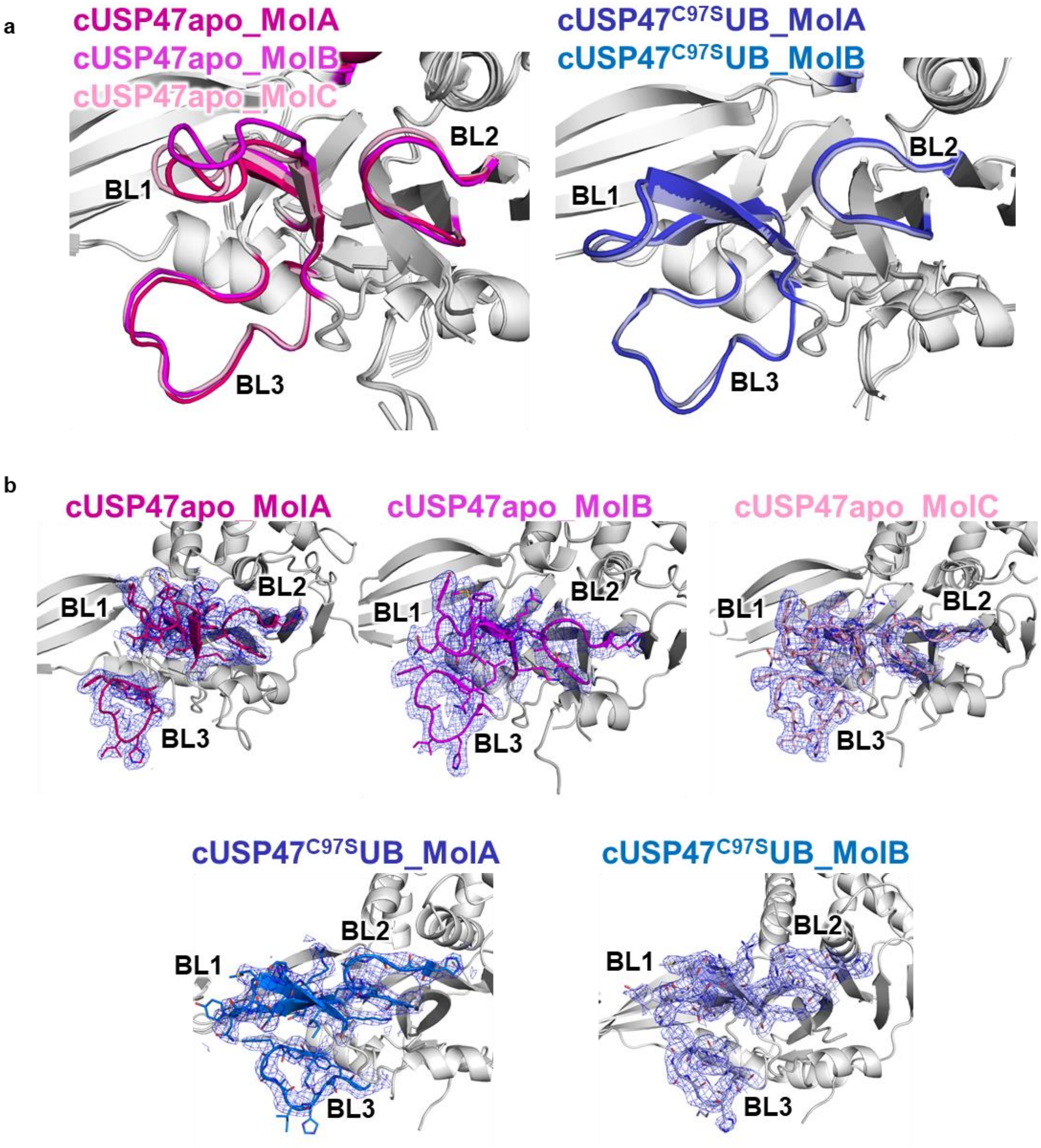

Supplementary Figure 7. Structural comparison of BL1, BL2, and BL3 of USP47$_{\mathrm{CD}}$.

(a) Superposition of crystallographically independent molecules in the *c*USP47$_{\mathrm{CD}}$ and cUSP47$^{\mathrm{C97S}}$:Ub structures. The BL1, BL2, and BL3 are highlighted. **(b)** The electron density maps for the BL1, BL2, and BL3 are shown for the three molecules in the *c*USP47$_{\mathrm{CD}}$ and *c*USP47$^{\mathrm{C97S}}$:Ub structures. The 2*F*o-*F*c electron density maps are contoured at 1σ level.

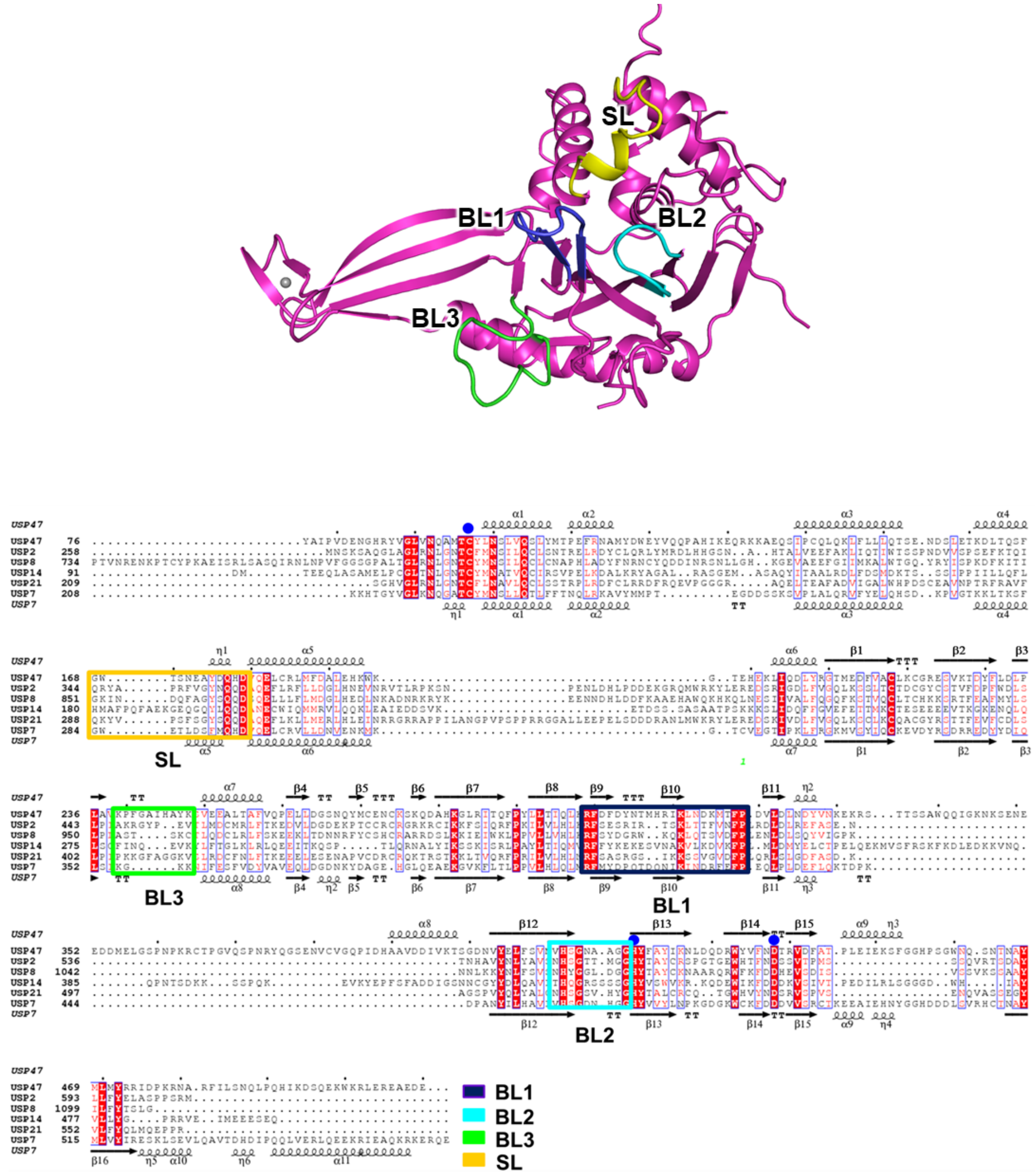

Supplementary Figure 8. Sequence alignment of the catalytic domain of USP47 with other USPs.

(a) Ribbon model of USP47. The SL, BL1, BL2, and BL3 are highlighted in yellow, blue, cyan, and green, respectively. **(b)** Structure-based sequence alignment for the catalytic domain. USP47 (Q22240), USP7 (Q93009), USP2 (O75604), USP8 (P40818), USP14 (P54578), and USP21 (Q9UK80) aligned using ClustalX 3.0. The image was produced using ESPript 3.0 (http://www.espript.ibcp.fr). The entry codes in the Swiss Prot database are in (). Except for the USP47, which is *C. elegans*, the sequences are of humans. Secondary structures for *c*USP47 and *h*USP7 are shown on the top and bottom, respectively.

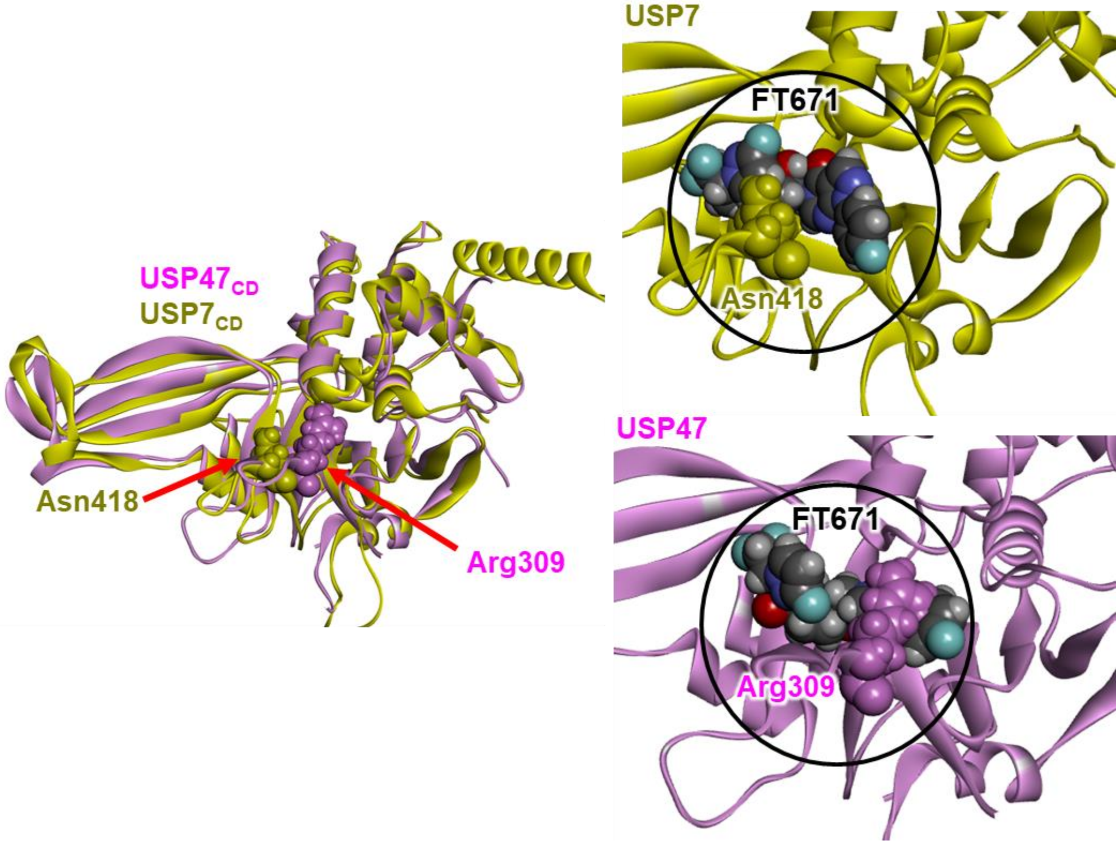

Supplementary Figure 9. Superposition of the apo *c*USP47 onto the FT671-bound USP7.

Superposition of the apo *c*USP47 (pink) structure onto the FT671-USP7 complex structure (yellow; PDB code: 5NGE) shows that FT671 makes unfavorable bumps with Arg309 of USP47 while the equivalent Asn418 of USP7 makes van der Waals interactions.

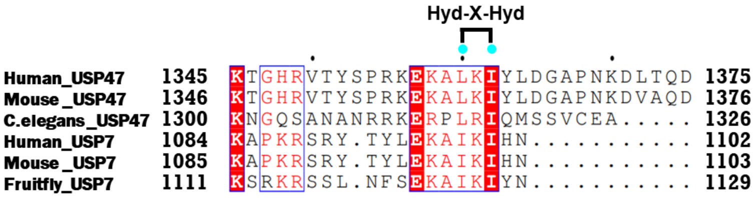

Supplementary Figure 10. Sequence alignment of the C-terminal region of USP47s and USP7.

Sequence alignment for the C-terminal tail of USP47s and USP7s. The sequence of USP47s from human (Q96K76), mouse (Q8BY87), and *C. elegans* (Q22240) and USP7s from human (Q93009), mouse (Q6A4J8), fruit fly (Q9VYQ8) were aligned using ClustalX 3.0, and the image was produced using ESPript 3.0 (http://www.espript.ibcp.fr). The entry codes in the Swiss Prot database are given in ().

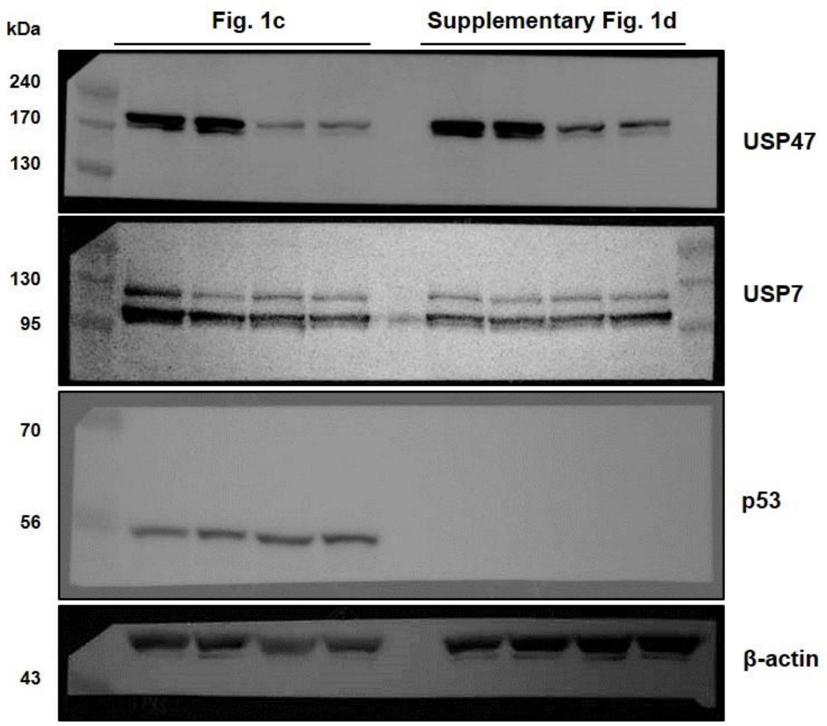
kDa
Fig. 1c
Supplementary Fig. 1d
240
170
130
USP47
130
95
USP7
70
56
p53
43
β-actin

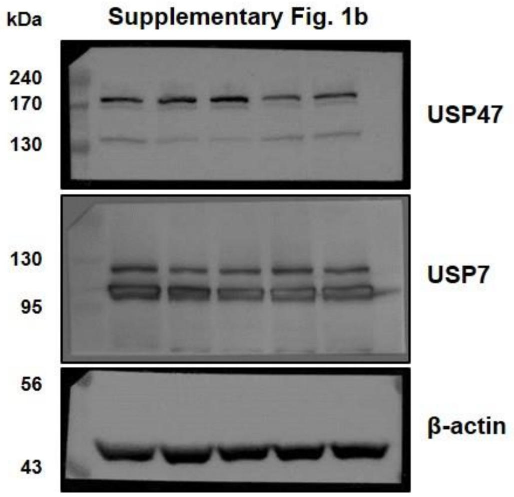
kDa
Supplementary Fig. 1b
240
170
130
USP47
130
95
USP7
56
43
β-actin

Fig. 2b

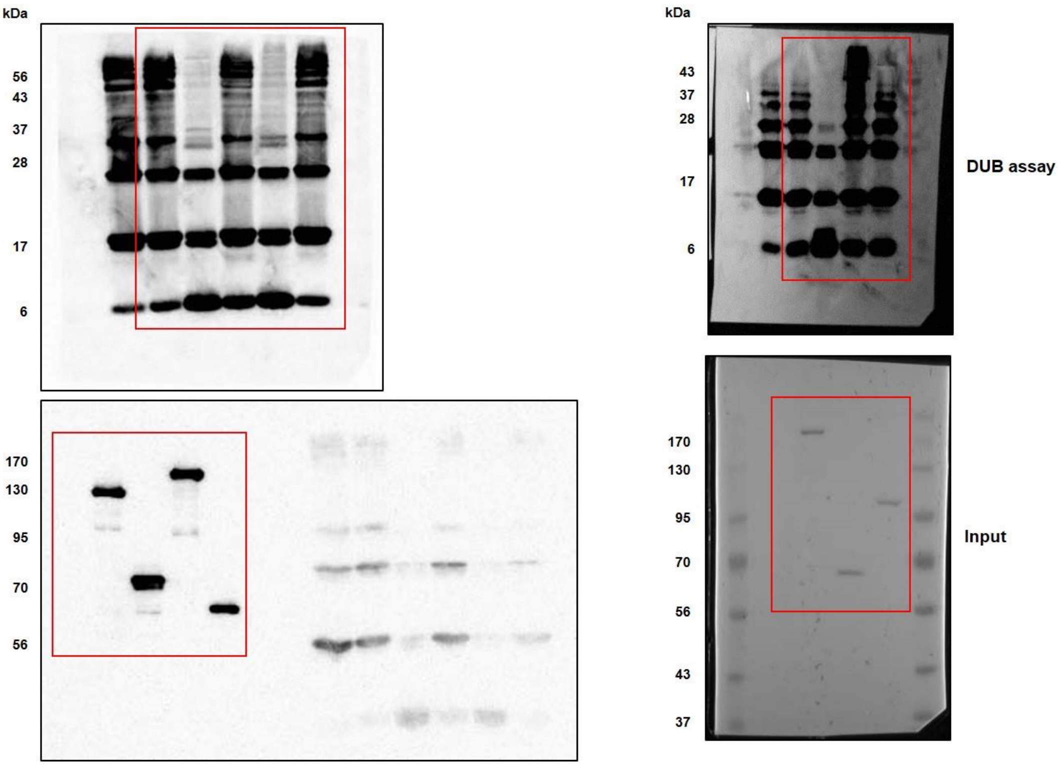
kDa
56
43
37
28
17
6
170
130
95
70
56
kDa
43
37
28
17
6
DUB assay
170
130
95
70
56
43
37
Input

Fig. 2f

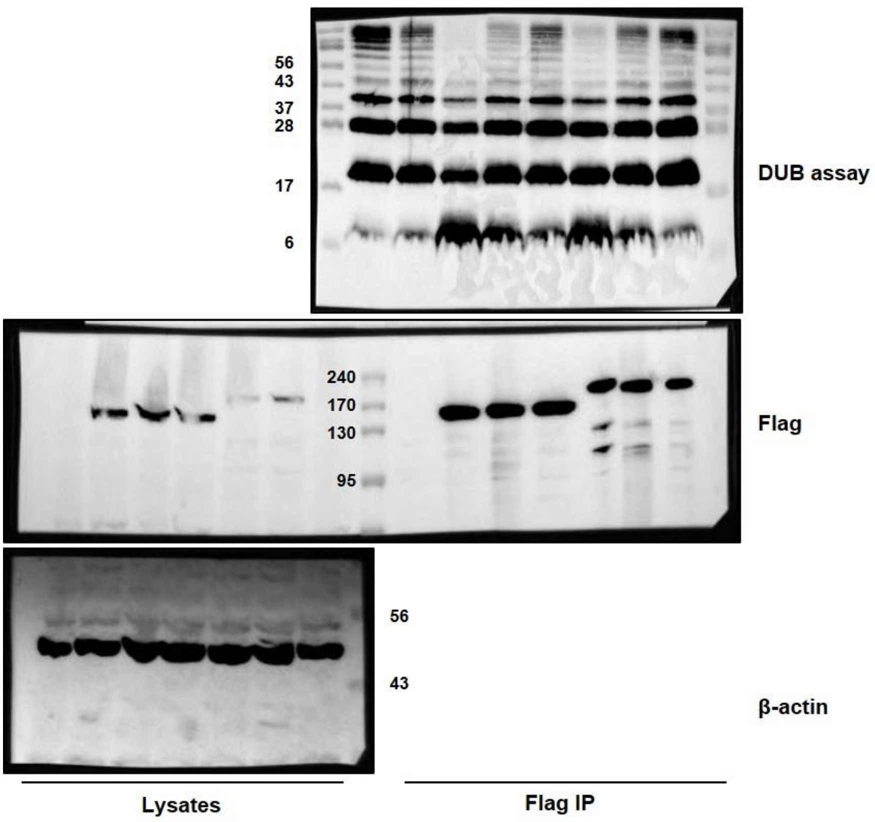
kDa
56
43
37
28
17
6
DUB assay
240
170
130
95
Flag
56
43
β-actin
Lysates
Flag IP

Fig. 1d

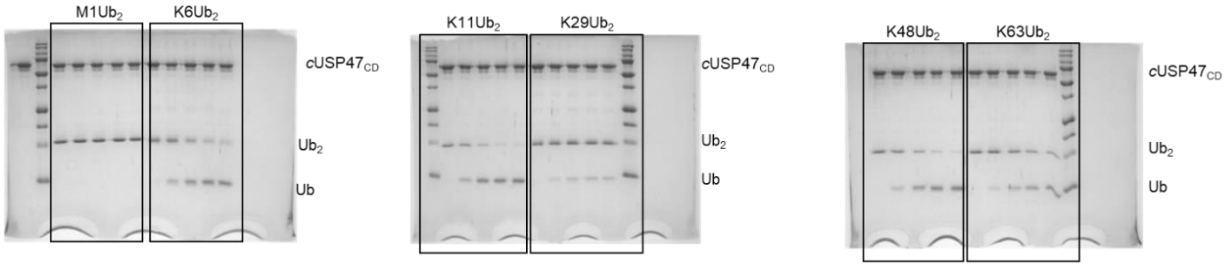

Fig. 6c

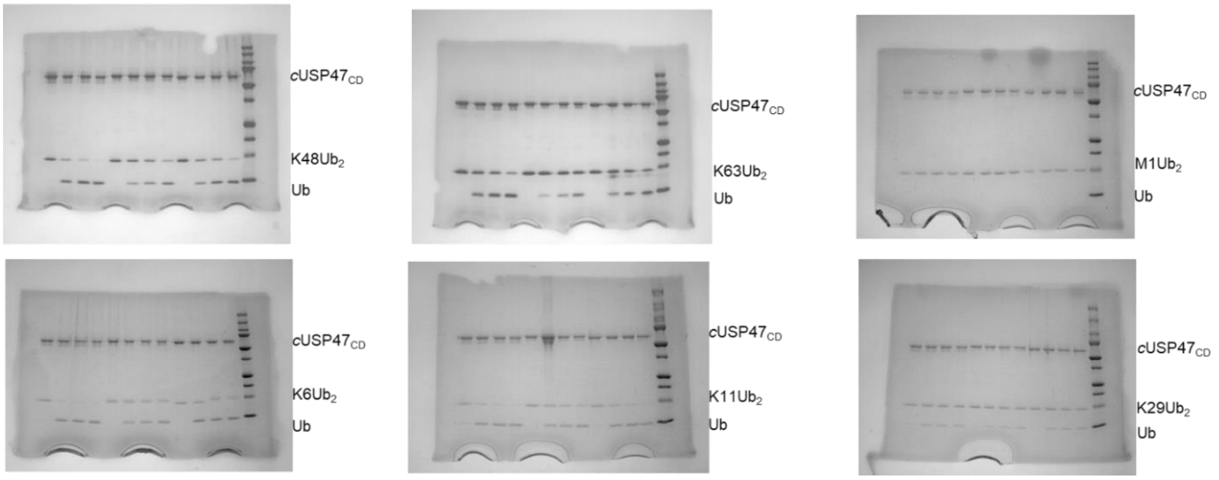

Supplementary Figure 11. Uncropped membranes used in this study**.**
