# Supplementary material for: Structural and functional characterization of USP47 reveals a hot spot for inhibitor design
Source: Commun Biol. 2023 Sep 22;6:970. doi: 10.1038/s42003-023-05345-5 (PMC10516900; doi:10.1038/s42003-023-05345-5)
Supplement: Supplementary file 2 — Description of Additional Supplementary Files [file 42003_2023_5345_MOESM2_ESM.pdf]

Description of Additional Supplementary Files

File name: Supplementary Data 1
Description: Source Data for Figures 1a, 1b, 1d, 1e, 1f, 2c, 2g, 3b, 4b, 4c, 4d, 5d, and 6b, and Supplementary Figures 1a and 1c.
