# Supplementary material for: Structural and functional characterization of USP47 reveals a hot spot for inhibitor design
Source: Commun Biol. 2023 Sep 22;6:970. doi: 10.1038/s42003-023-05345-5 (PMC10516900; doi:10.1038/s42003-023-05345-5)
Supplement: Supplementary file 4 — Reporting Summary [file 42003_2023_5345_MOESM4_ESM.pdf]

nature portfolio

Corresponding author(s): Eunice EunKyeong Kim

Last updated by author(s): Aug 28, 2023

Reporting Summary

Nature Portfolio wishes to improve the reproducibility of the work that we publish. This form provides structure for consistency and transparency in reporting. For further information on Nature Portfolio policies, see our Editorial Policies and the Editorial Policy Checklist.

Statistics

For all statistical analyses, confirm that the following items are present in the figure legend, table legend, main text, or Methods section.

n/a	Confirmed	
☐	☒	The exact sample size (*n*) for each experimental group/condition, given as a discrete number and unit of measurement
☐	☒	A statement on whether measurements were taken from distinct samples or whether the same sample was measured repeatedly
☒	☐	The statistical test(s) used AND whether they are one- or two-sided<br>*Only common tests should be described solely by name; describe more complex techniques in the Methods section.*
☒	☐	A description of all covariates tested
☒	☐	A description of any assumptions or corrections, such as tests of normality and adjustment for multiple comparisons
☐	☒	A full description of the statistical parameters including central tendency (e.g. means) or other basic estimates (e.g. regression coefficient) AND variation (e.g. standard deviation) or associated estimates of uncertainty (e.g. confidence intervals)
☒	☐	For null hypothesis testing, the test statistic (e.g. *F*, *t*, *r*) with confidence intervals, effect sizes, degrees of freedom and *P* value noted<br>*Give P values as exact values whenever suitable.*
☒	☐	For Bayesian analysis, information on the choice of priors and Markov chain Monte Carlo settings
☒	☐	For hierarchical and complex designs, identification of the appropriate level for tests and full reporting of outcomes
☒	☐	Estimates of effect sizes (e.g. Cohen's *d*, Pearson's *r*), indicating how they were calculated

Our web collection on statistics for biologists contains articles on many of the points above.

Software and code

Policy information about availability of computer code

Data collection	HKL2000 program (Otwinowski Z et al . 1997) was used for diffraction data collection
Data analysis	PHENIX program suite (v1.16-3549) for structure refinement; Pymol (v0.99rc6) for structure visualization;OriginPro(v9.0) for spectra visualization; Coot(v0.96) for structure refinement;Prims (v7.03) for measurement of enzyme activity.

For manuscripts utilizing custom algorithms or software that are central to the research but not yet described in published literature, software must be made available to editors and reviewers. We strongly encourage code deposition in a community repository (e.g. GitHub). See the Nature Portfolio guidelines for submitting code & software for further information.

Data

Policy information about availability of data

All manuscripts must include a data availability statement. This statement should provide the following information, where applicable:

- Accession codes, unique identifiers, or web links for publicly available datasets
- A description of any restrictions on data availability
- For clinical datasets or third party data, please ensure that the statement adheres to our policy

All are included in the text. X-ray crystallography data have been deposited in the RCSB protein data bank (PDB) with PDB codes: 7VE8(Crystal structure of USP47 catalytic domain), 7VE9 (Crystal structure of USP47 catalytic domain complex with ubiquitin).

Human research participants

Policy information about studies involving human research participants and Sex and Gender in Research.

Reporting on sex and gender	The work did not include human research.
Population characteristics	*Describe the covariate-relevant population characteristics of the human research participants (e.g. age, genotypic information, past and current diagnosis and treatment categories). If you filled out the behavioural & social sciences study design questions and have nothing to add here, write "See above."*
Recruitment	*Describe how participants were recruited. Outline any potential self-selection bias or other biases that may be present and how these are likely to impact results.*
Ethics oversight	*Identify the organization(s) that approved the study protocol.*

Note that full information on the approval of the study protocol must also be provided in the manuscript.

Field-specific reporting

Please select the one below that is the best fit for your research. If you are not sure, read the appropriate sections before making your selection.

☒ Life sciences ☐ Behavioural & social sciences ☐ Ecological, evolutionary & environmental sciences

For a reference copy of the document with all sections, see nature.com/documents/nr-reporting-summary-flat.pdf

Life sciences study design

All studies must disclose on these points even when the disclosure is negative.

Sample size	Statistical methods were not used to determine sample size, but were used to calculate standard error bar.
Data exclusions	No data was excluded.
Replication	All assay were typically performed at least in triplicate (n=3) to ascertain accurate value for the data shown.
Randomization	The samples were not allocated into different groups, therefor randomization was not required.
Blinding	Assays were performed non-blinded as this procedure is not applicable to biochemical studies of protein function.

Reporting for specific materials, systems and methods

We require information from authors about some types of materials, experimental systems and methods used in many studies. Here, indicate whether each material, system or method listed is relevant to your study. If you are not sure if a list item applies to your research, read the appropriate section before selecting a response.

Materials & experimental systems

n/a	Involved in the study	
☐	☒	Antibodies
☐	☒	Eukaryotic cell lines
☒	☐	Palaeontology and archaeology
☒	☐	Animals and other organisms
☒	☐	Clinical data
☒	☐	Dual use research of concern

Methods

n/a	Involved in the study	
☒	☐	ChIP-seq
☒	☐	Flow cytometry
☒	☐	MRI-based neuroimaging

Antibodies

Antibodies used	Primary antibodies: USP7- Bethyl Laboratories (Cat#A300-034), 1: 1000; USP47- Bethyl Laboratories (Cat#A31-048A), 1:1000; Flag-Sigma(Cat#F1804), 1:1000; Ubiquitin-Cell Signaling (Cat#3933), 1:2000; HSP90α/β- Santa Cruz Biotechnology (Cat#sc-13119), 1:5000 ; β-actin- AbFrontier (Cat#LF-PA0207), 1:5000

Validation	All antibodies are validated for the application of Western Blotting on human protein as per statements on the manufacturers' websites. Antibodies for USP47 and USP7 were further validated by RNA interference.

Eukaryotic cell lines

Policy information about cell lines and Sex and Gender in Research

Cell line source(s)	All cell lines were purchased from Korean cell line bank. (KCLB)
Authentication	All cell lines used in study have been authenticated by Korean cell line bank. (KCLB)
Mycoplasma contamination	All cell lines were confirmed to be free of mycoplasma contamination prior to ues.
Commonly misidentified lines (See ICLAC register)	No commonly misidentified lines were used.
